# Summer Epiphytic Diatoms from Terra Nova Bay and Cape Evans (Ross Sea, Antarctica) - A Synthesis and Final Conclusions

**DOI:** 10.1371/journal.pone.0153254

**Published:** 2016-04-14

**Authors:** Roksana Majewska, Peter Convey, Mario De Stefano

**Affiliations:** 1 Department of Environmental, Biological and Pharmaceutical Sciences and Technologies, II University of Naples, 81100, Caserta, Italy; 2 BioNEM Laboratory, Department of Experimental and Clinical Medicine, University “Magna Græcia” of Catanzaro, 88100, Catanzaro, Italy; 3 British Antarctic Survey, Natural Environment Research Council, High Cross, Madingley Road, Cambridge, CB3 0ET, United Kingdom; University of Waikato (National Institute of Water and Atmospheric Research), NEW ZEALAND

## Abstract

Despite recent advances in polar marine biology and related fields, many aspects of the ecological interactions that are crucial for the functioning of Antarctic shallow water habitats remain poorly understood. Although epiphytic diatoms play an essential role in the Antarctic marine food web, basic information regarding their ecology, biodiversity and biogeography is largely unavailable. Here, we synthesise studies on Ross Sea epiphytic diatoms collected during 11 summer Antarctic expeditions between the years 1989/90 and 2011/12, presenting a full list of diatom taxa associated with three macroalgal species (*Iridaea cordata*, *Phyllophora antarctica*, and *Plocamium cartilagineum*) and their epiphytic sessile fauna. Diatom communities found during the three summer months at various depths and sampling stations differed significantly in terms of species composition, growth form structure and abundances. Densities ranged from 21 to >8000 cells mm^-2^, and were significantly higher on the surface of epiphytic micro-fauna than on any of the macroalgal species examined. Generally, host organisms characterized by higher morphological heterogeneity (sessile microfauna, ramified *Plocamium*) supported richer diatom communities than those with more uniform surfaces (*Iridaea*). Differences between epiphytic communities associated with different macroalgae were reflected better in species composition than in growth form structure. The latter changed significantly with season, which was related strongly to the changing ice conditions. A general trend towards an increasing number of erect forms in deeper waters and tube-dwelling diatoms in the shallowest sites (2–5 m) was also observed. This study explores further important and largely previously unknown aspects of relationships and interactions between Antarctic epiphytic diatoms and their micro- and macro-environments.

## Introduction

Macroalgae are an important element of the shallow-water ecosystems of the Ross Sea. They provide refuge and habitats for micro- and macrofaunal communities and comprise a large biomass. Undoubtedly they constitute a significant food source for Antarctic marine organisms, but with most of this flowing through detritus-based food chains [1, 2, 3, 4, 5]. By this means, mineral nutrients are released into the water and sediments and can be assimilated by aquatic primary producers and incorporated again into the trophic system [6, 7]. Many herbivorous species also find both shelter and appropriate food source within the dense macroalgal beds, taking advantage of the rich diatom communities that often cover all submerged surfaces, including various parts of macroalgal thalli. There is growing evidence that a large proportion of the Antarctic herbivorous fauna feed mainly on benthic diatoms, which constitute a high quality and readily available food source [8, 9, 2, 10, 11, 12, 13, 14, 15, 16]. However, although epiphytic diatom communities clearly must play a particularly important role in the functioning of Antarctic shallow water ecosystems, many aspects of their ecology, taxonomy, distribution and biodiversity remain understudied and poorly understood.

Epiphytic diatom species are rarely reported in paleoenvironmental studies although they are often found in Antarctic marine sediments [17, 18, 19, 20, 21]. There is currently a pressing need for taxonomic and ecological characterization of polar diatoms for application in both paleoenvironmental and monitoring studies [22, 23, 24, 25]. However, for the generally unknown and unidentified Antarctic benthic species, environmental roles and significance cannot yet be established and, thus, the utility of benthic diatoms in paleoecological studies is currently limited [26, 27]. Therefore, detailed investigation of the contemporary distribution and diversity of Ross Sea epiphytic diatom communities and the factors controlling these is important to the understanding and proper interpretation of modern and past variations in the benthic diatom flora of this region.

**Fig 1:**
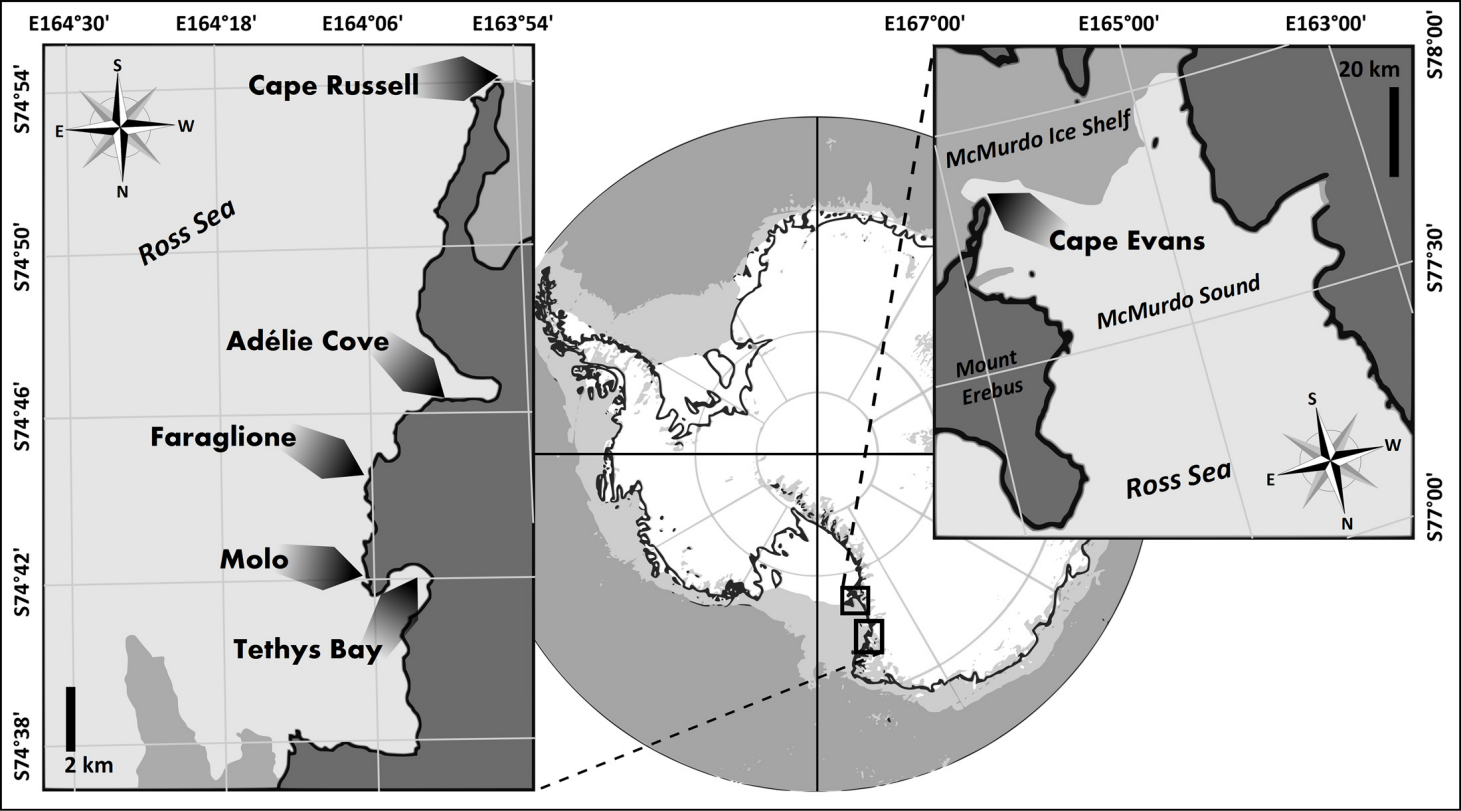
Study area and sampling sites.

This study builds on the results of a recent survey of marine epiphytic diatoms from the Ross Sea [28, 29, 30, 31]. We focus on epiphytic diatoms from Terra Nova Bay and Cape Evans (McMurdo Sound; Fig 1) examining material obtained during 11 summer Antarctic expeditions to these locations in the seasons 1989/90–2011/12. No comparable studies of epiphytes from other locations in the Ross Sea are available. We present qualitative and quantitative assessments of various components of the marine epiphytic diatom communities in order to derive better understanding of the multiple interactions that occur between them and the local environment. Biological, physical and chemical factors may regulate abundance, distribution and species composition of diatom communities associated with marine macroalgae. Amongst these, host macroalgal morphology, sampling site location and depth, grazing pressure, stage of season and ice formation and thaw have been identified as important factors influencing the shallow water communities [28, 29, 30, 31, 32, 33]. Reliable description of basibiont-epibiont-environment interactions is difficult as some of the factors involved are as yet unknown, while the environmental parameters likely to control Antarctic diatom development are strongly and dynamically interrelated. Thus, assessing the influence of each of the selected environmental factors, we considered both their direct effect on the diatom communities and the more ambiguous and complex interactions occurring among all of the elements in the investigated system.

## Materials and Methods

All samples were obtained during different Italian Antarctic Expeditions organized in the framework of the Italian National Antarctic Program (it. PNRA) coordinated by the Ministry of Education, University and Research (MIUR) through three national bodies: the National Scientific Committee for Antarctica (CSNA), the National Research Council (CNR), and the National Agency for New Technologies, Energy and Sustainable Economic Development (ENEA). Through the Antarctic Treaty System (ATS), the countries active in Antarctica, including Italy, consult on the uses of a whole continent and comply fully with the ATS’s regulatory requirements. Thus, all aspects related to material collection during the Italian Antarctic expeditions are regulated by the Antarctic Treaty and international rules of Antarctic environmental protection. In the present studies, samples collected within Antarctic Specially Protected Area No 161 (ASPA 161) were used. Permits for access and sampling in this area were issued by the Italian Ministry of Foreign Affairs upon a thorough evaluation of the research project. Our study did not involve endangered or protected species.

### Sampling stations

Thalli of three common macroalgal species (*Iridaea cordata*, *Phyllophora antarctica*, *Plocamium cartilagineum*) were collected by SCUBA divers from regularly used sampling locations: Tethys Bay (74°41.923'S, 164°01.670'E), Molo (74°54.187'S, 163°55.185'E), Faraglione (74°43.048'S; 164°06.425'E), Adélie Cove (74°46.470'S, 163°59.328'E), Cape Russell (74°41.393'S, 164°6.946'E) [29, 30, 31], and Cape Evans (77°38.066'S, 166°24.847'E; Fig 1; Table 1).

**Table 1:** Description of sampling effort: number of samples, replicates and macroalgal surface examined in different months and at various sites within the Ross Sea. In addition, a proportional value of macroalgal surface covered by epiphytic sessile fauna is given.

	Number of taxa found (genera)	Number of samples (replicates)	Total surface examined (mm^2^)	Months	Associated fauna (% of surface)	Sampling sites*	Locations**
*Iridea cordata*	55 (26)	14 (42)	42	Dec, Jan, Feb	3.1	CR, AC, F, M, TB	TNB
*Phyllophora antarctica*	95 (39)	24 (100)	140	Dec, Jan, Feb	9.5	CR, AC, F, M, TB, CE	TNB, MS
*Plocamium cartilagineum*	60 (29)	8 (24)	39	Jan, Feb	6.8	CR, AC, F	TNB
In total	109 (44)	46 (166)	221	Dec, Jan, Feb	7.8	CR, AC, F, M, TB, CE	TNB, MS

*CR—Cape Russell, AD—Adélie Cove, F—Faraglione, M—Molo, TB—Tethys Bay, CE—Cape Evans

**TNB—Terra Nova Bay, MS—McMurdo Sound

### Sampling procedures

At Cape Evans samples were collected through holes made in the sea ice with a Reed drill [28] (for list of samples see S1 Table). Healthy thalli of similar size were carefully scraped off the rocks and placed in individual plastic bags. Immediately after collection, the material was fixed with 4% formaldehyde solution in filtered sea water. As we were interested in the entire surface-associated community (including both metaphytic and true epiphytic taxa), no additional cleaning procedures were applied before the fixation in formalin [29, 30].

### Microscopic analyses

For diatom counting and growth form analysis, ca. 1 cm^2^ subsamples were cut from each of the thalli collected. Macroalgal pieces were then dehydrated through a 25, 50, 60, 70, 80, 90, 95, 100% alcohol series, treated with a Critical Point Dryer (K850 EMITECH), placed on aluminium stubs and sputter-coated with gold-palladium or platinum using a DESK V HP TSC Cold Sputter Coater. For taxonomic examination, small sections (ca. 2 cm^2^) of macroalgal thalli were digested with boiling concentrated acid (64% nitric acid and 97% sulphuric acid in a 1:3 volume ratio), rinsed thoroughly with distilled water, centrifuged and decanted. Prior to SEM observations, clean material was mounted on aluminium stubs and sputter-coated with platinum. Diatoms were identified and enumerated on a surface area of ca. 1–2 mm^2^ of each of the 166 collected macroalgal individuals at magnifications ranging between 400x to 60000x using JEOL JSM 60/60 LW and Zeiss Supra 40 scanning electron microscopes. In addition, for the epizooic community analyses, 0.5 mm^2^ (or larger) samples of epiphytic sessile microfauna were examined for each *Phyllophora* and *Plocamium* thallus.

### Species classification

For structural and functional analysis of the communities, identified taxa were divided into growth form groups: erect (epiphytic cells attached to the substrate by stalks, pads or peduncles), adnate (cells strongly adhering to macroalgal surface), motile (biraphid cells moving on the substrate surface), tube-dwelling (cells producing mucilage tubes), planktonic (true pelagic species), and plocon (metaphytic cells loosely associated with the algal surface). The following literature was used to identify the diatom specimens: Ehrenberg [34], Van Heurck [35], Mangin [36], Peragallo [37], Heiden and Kolbe [38], Manguin [39, 40], Frenguelli and Orlando [41], Hustedt [42], Frenguelli [43], Hasle [44], Poulin *et al*. [45, 46, 47], Hasle *et al*. [48], Romero and Rivera [49], Cremer *et al*. [50], Scott and Thomas [51], Fernandes *et al*. [52], Al-Handal *et al*. [53, 54], Al-Handal and Wulff [55, 56], Cefarelli *et al*. [57], and Riaux-Gobin *et al*. [58].

### Statistical analyses

Statistical analyses were performed using Canoco v5 [59], EstimateS v9.1 [60] and Primer v6 [61] software. Analysis of similarities (ANOSIM) was used to determine whether significant differences in diatom community growth form structure and species composition occurred among the selected macroalgal species, sampling seasons, sites, and depths. Nonmetric multidimensional scaling (nMDS) ordination was used to display differences in communities associated with different macroalgal hosts. To estimate the similarity within groups and dissimilarity among them, and to indicate the percentage contribution of the most important species to the average inter-group dissimilarity, a similarity percentage analysis (SIMPER) was applied. To visualize the effects of selected factors on diatom communities, a redundancy analysis (RDA) and partial RDA were performed on log-transformed abundance data and biplot diagrams were drawn. A Monte Carlo permutation test was used to test the significance of the axes (4999 permutations, p < 0.05). In addition, taxa accumulation curves and estimated total species richness were computed using sample-based rarefaction and functional extrapolation methods proposed by Colwell *et al*. [60, 62].

## Results

### Diatom species composition

A total of 109 diatom species (44 genera) was found during the survey (Table 2). Three species, *Cocconeis fasiolata*, *Fragilariopsis nana* and *Navicula perminuta* occurred in all 46 samples (166 macroalgal replicates) analysed, and *Achnanthes brevipes*, *A*. *vicentii*, *C*. *antiqua*, *F*. *curta*, *Melosira adeliae*, *Pseudogomphonema kamtschaticum* and *Synedropsis recta* were present in more than 75% of the samples. Eight species (*Cocconeis antiqua*, *C*. *costata*, *C*. *fasciolata*, *F*. *nana*, *Melosira adeliae*, *Naviaula perminuta*, *Parlibellus delognei*, *Pseudogomphonema kamtschaticum*) contributed 25% of total diatom abundance. Of these, *Cocconeis antiqua* comprised over 50% of diatoms counted in at least one of the samples and *C*. *fasciolata* and *Navicula perminuta* contributed 75% of the total diatom numbers. Twenty-six taxa were found in only a single sample, and 65 species contributed less than 1% of the total diatom abundance across all samples (Table 2).

**Table 2:** Epiphytic diatoms recorded on three macroalgal host species (*Iridaea cordata*, *Phyllophora antarctica*, *Plocamium cartilagineum*) at six sampling sites during 11 Antarctic expeditions.

TAXA	% of total abundance	host macroalga*	Locations**	% of samples***			
				*Iridae cordata*	*Phyllophpra antarctica*	*Plocamium cartulagineum*	All samples
*Achnanthes brevipes* Agardh	<1–13.6	Ir, Ph, Pl	CR, AC, F, M, TB, CE	93	100	100	97.8
*Achnanthes* sp. 1	<1	Pl	F	0	0	37.5	6.5
*Achnanthes* sp. 2	<1	Ph, Pl	F	0	4.2	25	6.5
*Achnanthes* sp. 3	<1–13.7	Ph	AC, TB, CE	0	12.5	0	6.5
*Achnanthes vicentii* Manguin	<1–18.1	Ir, Ph, Pl	CR, AC, F, M, TB, CE	71.5	100	100	91.3
*Actinocyclus actinochilus* (Ehrenberg) Simonsen	<1	Ir	F	7.1	0	0	2.2
*Amphiprora kufferathii* Manguin	<1	Ph	AC, M, F	0	25	0	13
*Amphora* cf. *cymbelloides* Grunow	<1–2.6	Ph	TB, CE	0	8.3	0	4.3
*Amphora* cf. *racovitzae* Van Heurck	<1	Ir, Ph	F, M, CE	14.3	29.2	0	19.6
*Amphora cf*. *terroris Ehrenberg*	<1	Ph, Pl	AC, F, TB, CE	0	50	12.5	15.2
*Amphora racovitzae* Van Heurck	<1–1.4	Ir, Ph, Pl	CR, AC, F, M, TB	28.6	66.7	87.5	58.7
*Amphora* sp. 1	<1–10	Ir, Ph, Pl	AC, F, M, TB, CE	50	66.7	87.5	65.2
*Amphora* sp. 2	<1	Ir, Ph, Pl	AC, F, M, TB	14.3	29.2	12.5	21.7
*Amphora* type C (sensu Scott & Marchant, 2005)	<1	Ir, Ph	F, M	7.1	8.3	0	6.5
*Asteromphalus hookeri* Ehrenberg	<1	Ph	M	0	4.2	0	2.2
*Attheya gaussii* (Heiden) Crawford	<1	Ph, Pl	CR, AC, F, M	0	16.75	62.5	19.6
*Auricula compacta* (Hustedt) Medlin	<1	Ir, Ph, Pl	CR, AC, F, M, TB	28.6	29.2	37.5	30.4
*Brandinia mosimanniae* Fernandes & Procopiak	<1–1.6	Ir, Ph	AC, F	7.1	12.5	37.5	15.2
*Chaetoceros dichaeta* Ehrenberg	<1	Pl	F	0	0	12.5	2.2
*Chaetoceros neglectus* Karsten	<1–7	Pl	AC, F	0	0	62.5	20.8
*Chaetoceros* sp. 1	<1–2.4	Ph, Pl	AC, F	0	8.3	62.5	15.2
*Chaetoceros* sp. 2	<1–1.2	Ph	AC, F, TB	0	12.5	0	6.5
*Cocconeis antiqua* Tempère & Brun	<1–54.5	Ir, Ph, Pl	CR, AC, F, M	100	70.8	87.5	82.6
*Cocconeis californica* var. *kerguelensis* Heiden	<1	Ph	TB	0	4.2	0	2.2
*Cocconeis* cf. *californica* Grunow	<1	Ph	AC, F, M, TB	0	16.7	0	8.7
*Cocconeis* cf. *californica* sensu Al-Handal & Wulff [55]	<1–23.8	Ir, Ph, Pl	CR, AC, F, M, TB	14.3	16.7	75	26.1
*Cocconeis* cf. *costata* Gregory	<1	Ir	F	7.1	0	0	2.2
*Cocconeis* cf. *neothumensis* Krammer	<1	Ph	CE	0	4.2	0	2.2
*Cocconeis* cf. *stauroneiformis* (W. Smith) Okuno	<1–11.3	Ph, Pl	AC, F, M, TB, CE	0	25	75	26.1
*Cocconeis costata* Gregory var. *antarctica* Manguin	<1–42.6	Ir, Ph, Pl	AC, F, M	7.1	12.5	50	21.7
*Cocconeis fasciolata* (Ehrenberg) Brown	<1–78.8	Ir, Ph, Pl	CR, AC, F, M, TB, CE	100	100	100	100
*Cocconeis melchioroides* Al-Handal, Riaux-Gobin, Romero & Wulff	<1–12.2	Ph, Pl	F, AC	0	4.2	62.5	13
*Cocconeis* sp. 1 (sensu Al-Handal and Wulff [56])	<1	Ir, Ph	AC, M, CE	7.1	8.3	0	6.5
*Cocconeis* sp. 2 (sensu Majewska et al. [29])	<1–5.2	Ir, Ph, Pl	AC, F, M, TB	50	62.5	50	56.5
*Cocconeis* sp. 3 (sensu Majewska & De Stefano [28])	<1–2.6	Ph	TB, CE	0	8.3	0	4.3
*Cocconeis* sp. 4 (sensu Majewska et al. [31])	<1	Pl	F	0	0	12.5	2.2
*Coscinodiscus* sp.	<1	Ir	M	7.1	0	0	2.2
*Entomoneis* sp.	<1	Ph	F	0	8.3	0	4.3
Eunotioid	<1	Ph	CE	0	4.2	0	2.2
*Eunotogramma marginopunctatum* Long, Fuge & Smith	<1	Ph	CE	0	4.2	0	2.2
*Fallacia marnieri* (Manguin) Witkowski, Lange-Bertalot & Metzeltin	<1–1.5	Ph, Pl	CR, AC, F, M, TB, CE	0	87.5	37.5	52.2
*Fragilaria* cf. *striatula* Lyngbye	<1–4.4	Ph, Pl	AC, F, TB	0	8.3	62.5	15.2
*Fragilaria islandica* var. *adeliae* Manguin	<1–1.4	Ir, Ph, Pl	AC, F, M, TB, CE	7.1	33.3	12.5	21.7
*Fragilaria* sp.1	<1–7.2	Ir, Ph	F, M, TB	14.3	29.2	0	19.6
*Fragilaria* sp.2	<1	Ph	CE	0	4.2	0	2.2
*Fragilariopsis curta* (Van Heurck) Krieger	<1–14.2	Ir, Ph, Pl	CR, AC, F, M, TB, CE	92.9	100	100	97.8
*Fragilariopsis cylindrus* (Grunow) Krieger	<1–1.6	Ir, Ph	TB, M, F	14.3	12.5	0	10.9
*Fragilariopsis kergulensis* (O'Meara) Hustedt	<1	Ph	TB, M	0	8.3	0	4.3
*Fragilariopsis nana* (Steemann Nielsen) Paasche	<1–34.2	Ir, Ph, Pl	CR, AC, F, M, TB, CE	100	100	100	100
*Fragilariopsis obliquecostata* (Van Heurck) Heiden	<1	Ir, Ph, Pl	CR, AC, F, M, TB	21.4	20.8	12.5	19.6
*Fragilariopsis rhombica* (O'Meara) Hustedt	<1	Ph	CR	0	4.2	0	2.2
*Fragilariopsis ritscheri* Hustedt	<1	Ir, Ph, Pl	AC, F, M, TB	7.1	12.5	25	13
*Fragilariopsis sublinearis* (Van Heurck) Heiden	<1	Ir, Ph, Pl	CR, AC, F, M, TB, CE	7.1	20.8	50	21.7
*Gomphonemopsis littoralis* (Hendey) Medlin	<1	Ph	CE	0	4.2	0	2.2
*Grammatophora arctica* Cleve	<1	Ir, Ph	F, CE	14.3	4.2	0	6.5
*Grammatophora arcuata* Ehrenberg	<1–1.5	Ir, Ph, Pl	AC, F, M, CE	7.1	20.8	25	17.4
*Gyrosigma* sp.	<1	Ir, Ph	AC	7.1	4.2	0	4.3
*Haslea trompii* (Cleve) Simonsen	<1	Ph	AC	0	4.2	0	2.2
*Hyalodiscus* sp.	<1	Pl	F	0	0	12.5	2.2
*Licmophora gracilis* (Ehrenberg) Grunow	<1	Pl	F	0	0	12.5	2.2
*Melosira adeliae* Manguin	<1–27.4	Ir, Ph, Pl	CR, AC, F, M, TB, CE	92.9	79.2	87.5	84.8
*Melosira moniliformis* var. *australis* (Peragallo) Manguin	<1	Ph, Pl	AC, F	0	4.2	12.5	4.3
*Melosira* sp.	<1	Ph	CE	0	4.2	0	2.2
*Navicula* cf. *criophila* (Castracane) De Toni	<1	Ph, Pl	F, CE	0	4.2	37.5	8.7
*Navicula* cf. *gelida* Grunow	<1	Ir, Ph	F, M	7.1	4.2	0	4.3
*Navicula* cf. *incertata* Lange-Bertalot & Krammer	<1–6	Ir, Ph	CR, AC, F, M, TB	50	75	0	54.3
*Navicula* cf. *jejunoides* Van Heurck	<1–6.7	Ir, Ph, Pl	CR, AC, F, M, TB, CE	42.9	83.3	75	69.6
*Navicula directa* (W Smith) Ralfs	<1	Ir, Ph, Pl	F, M, TB, CE	14.3	25	12.5	19.6
*Navicula glaciei* Van Heurck	<1–11.3	Ir, Ph, Pl	CR, AC, F, M, TB, CE	50	79.2	75	69.6
*Navicula perminuta* Grunow	5.8–75.4	Ir, Ph, Pl	CR, AC, F, M, TB, CE	100	100	100	100
*Navicula* sp. 1	<1	Ph	F, CE	0	8.3	12.5	6.5
*Navicula* sp. 2	<1	Ph	CE	0	4.2	0	2.2
*Navicymbula* sp.	<1–1.7	Pl	AC, F	0	8.3	62.5	15.2
*Nitzschia acicularis* (Kützing) W. Smith	<1	Ph, Pl	AC, F, M	0	12.5	37.5	13
*Nitzschia* cf. *australis* (M. Peragallo) A. Mann	<1	Pl	F	0	0	12.5	2.2
*Nitzschia* cf. *lecointei* Van Heurck	<1–2.7	Ir, Ph, Pl	AC, F, M, TB, CE	14.3	45.8	62.5	39.1
*Nitzschia* cf. *palea* (Kützing) W. Smith	<1–5.5	Pl	AC, F	0	0	62.5	10.9
*Nitzschia medioconstricta* Hustedt	<1–2.5	Ir, Ph, Pl	AC, F, M, TB	42.9	75	75	65.2
*Nitzschia* sp. 1	<1	Ir, Ph	CR, AC, F, M, CE	21.4	25	0	19.6
*Nitzschia* sp. 2	<1	Ph	CE	0	4.2	0	2.2
*Nitzschia stellata*	<1–2.4	Ir, Ph	CR, AC, F, M, TB, CE	42.9	70.8	0	50
*Odontella litigiosa* (Van Heurck) Hoban	<1–1.6	Ir, Ph, Pl	AC, F, M, TB, CE	7.1	12.5	37.5	15.2
*Paralia sol* (Ehrenberg) Crawford	<1–1.2	Ir, Ph, Pl	CR, AC, F, M	7.1	16.7	25	15.2
*Parlibellus delognei* (Van Heurck) Cox	<1–41.2	Ir, Ph	AC, F, M, TB	57.1	75	0	56.2
*Pinnularia quadratarea* (Schmidt) Cleve	<1	Ph	AC, F, TB	0	12.5	0	6.5
*Planothidium* cf. *dubium* (Grunow) Round & Bukhtiyarova	<1	Pl	AC	0	0	12.5	2.2
*Planothidium* sp.	<1	Ph	TB	0	4.2	0	2.2
*Pleurosigma directum* Grunow	<1	Ir, Ph, Pl	CR, AC, F, M, TB	14.3	16.7	12.5	15.2
*Pleurosigma* sp.	<1	Ph	CE	0	4.2	0	2.2
*Podosira* sp.	<1–1.3	Ir, Ph, Pl	CR, AC, F, M, TB	14.3	33.3	35	4.3
*Porosira glacialis* (Grunow) Jørgensen	<1	Ir, Ph, Pl	F, M, TB, CE	7.1	37.5	62.5	32.6
*Porosira pseudodenticulata* (Hustedt) Jousé	<1	Ph	AC, F, TB	0	12.5	0	6.5
*Pseudogomphonema kamtschaticum* (Grunow) Medlin	<1–32.1	Ir, Ph, Pl	CR, AC, F, M, TB, CE	100	95.8	87.5	95.7
*Pseudonitzschia* sp.	<1	Ph, Pl	AC, F, M	0	12.5	12.5	8.7
*Pseudostaurosira brevistriata* (Grunow) DM Williams & Round	<1	Ph, Pl	AC, F, TB, CE	0	25	37.5	17.4
*Rhizosolenia* sp.	<1	Ph	TB	0	4.2	0	2.2
*Synedropsis fragilis* (Manguin) Hasle, Syvertsen & Medlin	<1	Pl	F	0	0	12.5	2.2
*Synedropsis hyperboreoides* Hasle, Medlin & Syvertsen	<1	Ir, Ph	AC, F, M	7.1	8.3	0	6.5
*Synedropsis leavis* (Heiden) Hasle, Medlin & Syvertsen	<1	Ph	AC	0	4.2	0	2.2
*Synedropsis recta* Hasle, Syvertsen & Medlin	<1–4.5	Ir, Ph, Pl	CR, AC, F, M, TB, CE	92.9	100	75	93.5
*Tabularia tabulata* (Agardh) Snoeijs	<1–8.6	Ir, Ph, Pl	AC, F, M, TB, CE	14.3	45.8	62.5	39.1
*Thalassiosira antarctica* Comber	<1	Ir, Ph	F, M	7.1	8.3	0	6.5
*Thalassiosira* cf. *ambigua* Kozlova	<1	Ir, Ph	AC, F, M	14.3	8.3	0	8.7
*Thalassiosira gracilis* (Karsten) Hustedt	<1	Ph	M, TB	0	16.7	0	8.7
*Thalassiosira* sp. 1	<1	Ir, Ph, Pl	AC, F, M	14.3	8.3	12.5	10.9
*Thalassiothrix antarctica* Schimper ex Karsten	<1	Ph	CR, M	0	8.3	0	4.3
*Thalassiothrix longissima* Cleve & Grunow	<1	Ph	M, TB	0	8.3	0	4.3
*Trachyneis aspera* (Ehrenberg) Cleve	<1	Ir, Ph, Pl	CR, AC, F, M, TB, CE	14.3	58.3	37.5	41.3
*Trigonium arcticum* (Brightwell) Cleve	<1–4.1	Ir, Ph, Pl	CR, AC, F, M, TB	42.9	29.2	100	45.7

*Ir—*Iridaea cordata*, Ph—*Phyllophora antarctica*, Pl—*Plocamium cartilagineum*

**CR—Cape Russell, AD—Adélie Cove, F—Faraglione, M—Molo, TB—Tethys Bay, CE—Cape Evans

***% of samples in which the taxon was found

### Diatom communities and environmental factors

ANOSIM indicated that the diatom communities differed significantly among different sampling seasons, sites, and depths in terms of both species composition and growth form structure. The highest Global R (0.485) value was obtained in the analysis of similarity among diatom communities associated with different macroalgal species when growth form was considered, indicating a high separation between the groups. However, the same test performed on species data indicated that the difference between communities on different macroalgal hosts was not significant (p>0.05; Table 3). Growth form structure appeared to be a parameter that better reflected differences among communities when testing the influence of season and depth, but not that of sampling site (Table 3).

**Table 3:** Results of ANOSIM test performed on species and growth form abundance data.

	Host alga		Season		Sampling site		Depth	
	GF	S	GF	S	GF	S	GF	S
*p*	>0.05	0.01	0.005	0.01	0.01	0.005	0.005	0.01
Global R	0.098	0.485	0.389	0.317	0.231	0.319	0.309	0.215

GF—growth form, S—species

### Host macroalga

**Fig 2:**
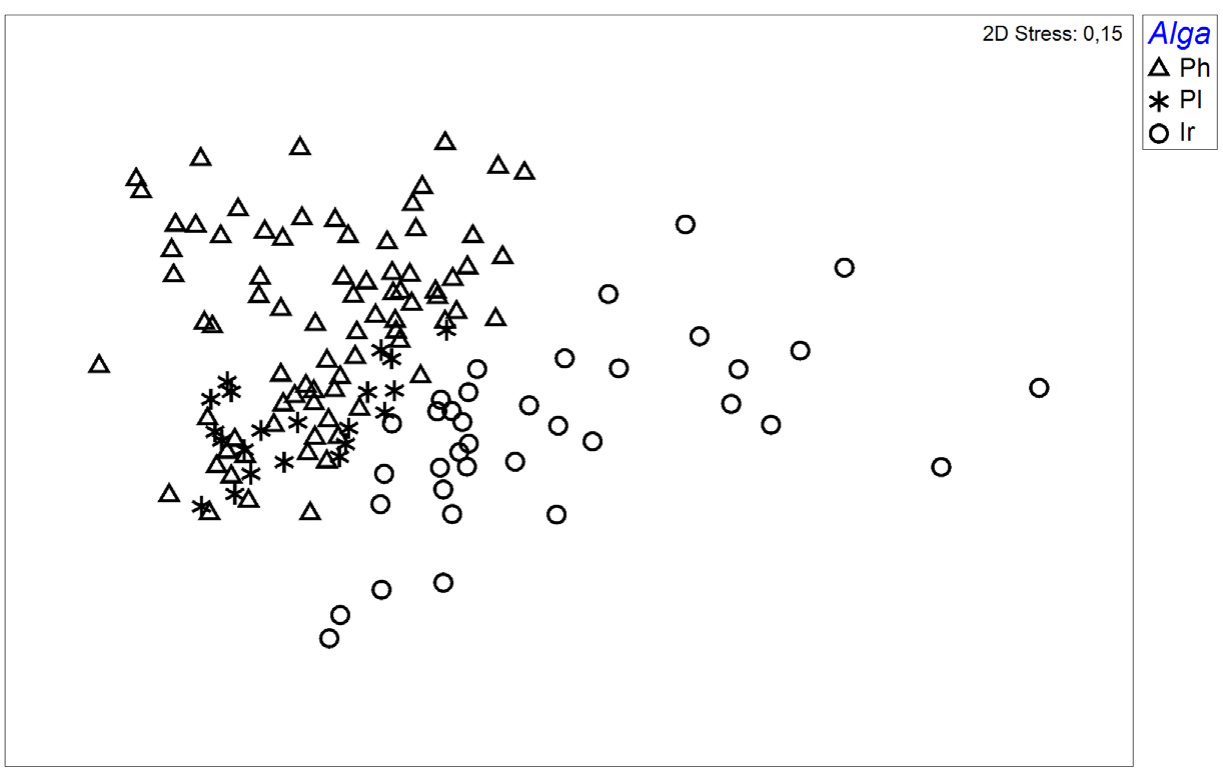
Nonmetric multidimensional scaling (nMDS) graph based on the species abundance data. Ph—samples of epiphytic diatom communities associated with *Phyllophora antarctica*, Pl—samples of epiphytic diatom communities associated with *Plocamium cartilagineum*, Ir—samples of epiphytic diatom communities associated with *Iridaea cordata*.

The nMDS performed on species abundance data revealed differences among diatom communities associated with different macroalgal hosts (Fig 2). A relatively low stress value (0.15) indicated that the 2-dimensional solution was sufficient to obtain a reliable result. As indicated by SIMPER analysis, average similarity within groups was 41%, 47%, and 57.8% for *Iridaea*, *Phyllophora*, and *Plocamium* epiphytes, respectively. The level of dissimilarity between groups ranged from 53.7% (*Phyllophora* vs *Plocamium*) to 67% (*Phyllophora* vs *Iridaea*). Seven species: *Achnanthes vincentii*, *Coconeis antiqua*, *C*. *fasciolata*, *Fragilariopsis nana*, *Navicula perminuta*, *Pseudogomphonema kamtschaticum*, and *Tabularia tabulata* were essential for the group differentiation (S2–S4 Tables).

**Fig 3:**
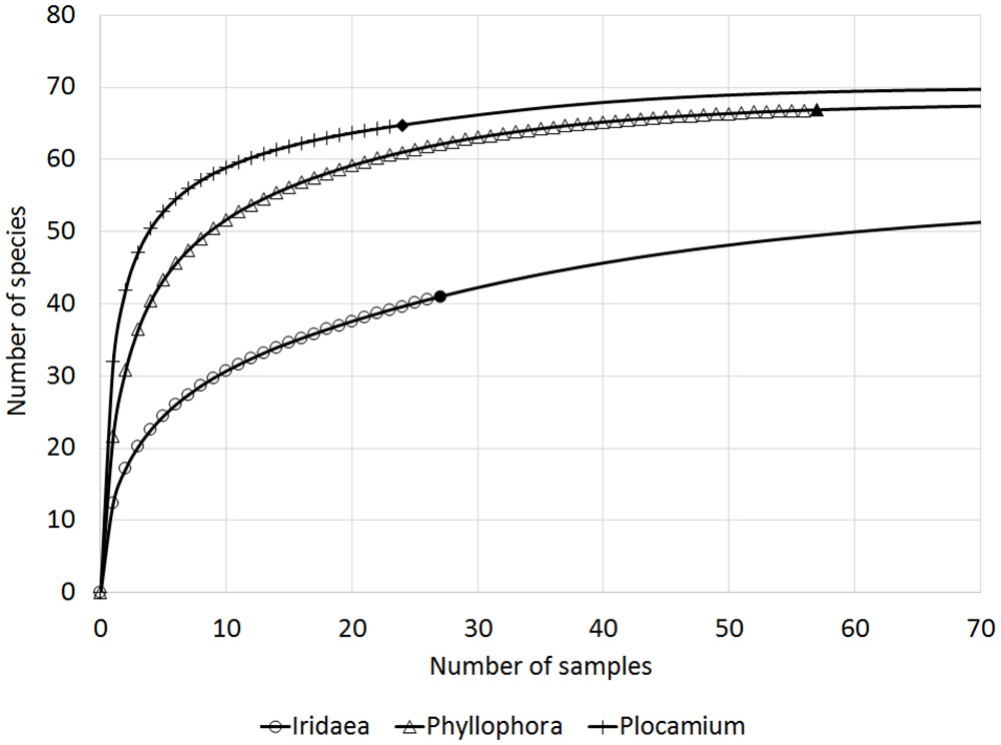
Extrapolation (plain lines) of rarefaction curves (lines with symbols) based on samples from locations where all three algal species were found (Cape Russell, Adélie Cove, Faraglione).

The highest number of diatom taxa was found associated with thalli of *Phyllophora* (95), followed by *Plocamium* (60) and *Iridaea* (55). It should be noted, however, that the numbers of samples examined, replicates and the examined macroalgal surface, as well as the period of study and study locations, differed for different host species (Table 1). Therefore, to equalize the information content of each of the sample sets, rarefaction and extrapolation curves were computed (Fig 3). The analyses indicated that *Plocamium* may be the host algal species that supports the richest diatom community.

### Seasons

**Fig 4:**
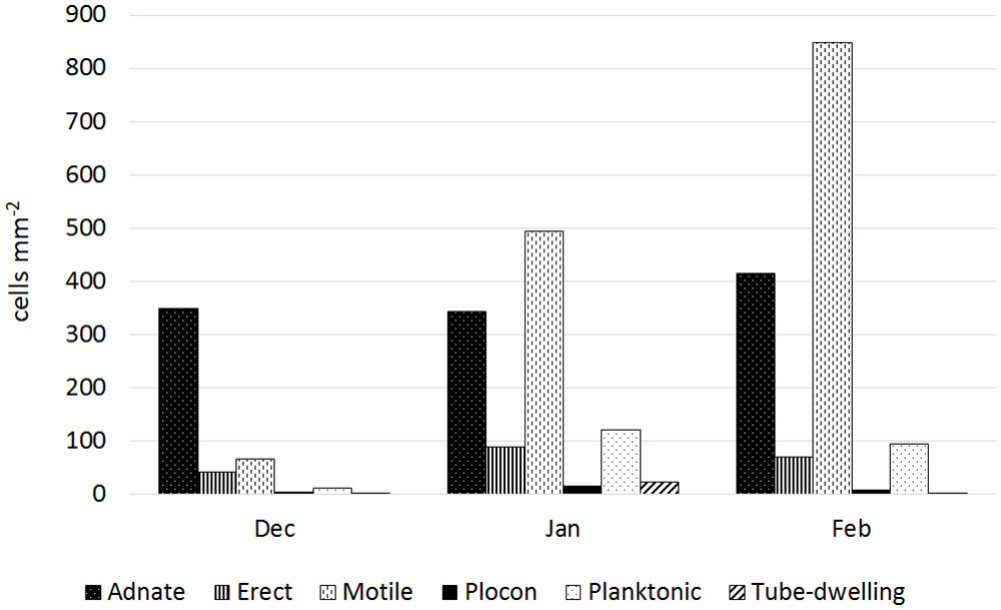
Average abundances of diatom growth forms found on macroalgal surface in different months (based on all macroalgal replicates collected in Terra Nova Bay).

**Fig 5:**
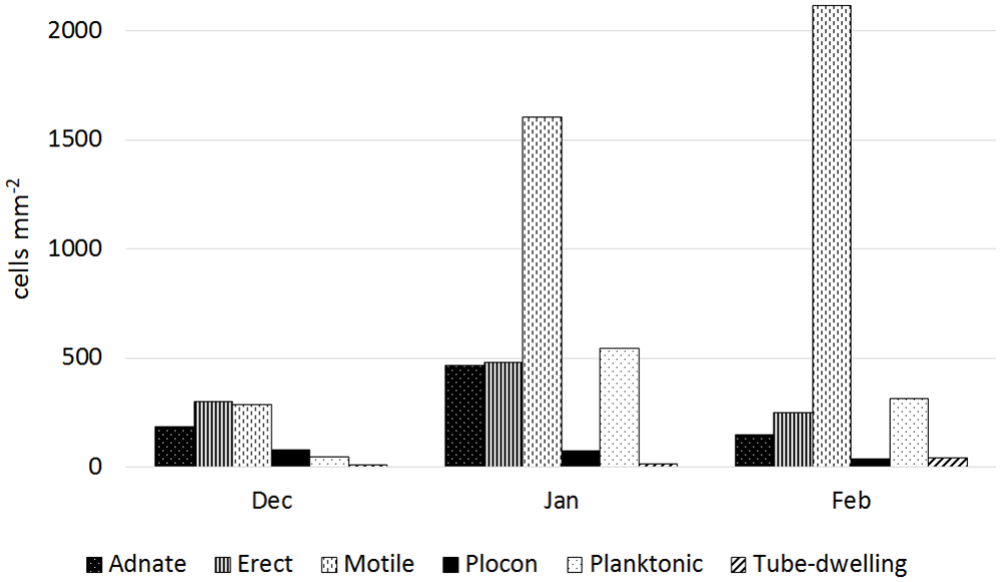
Average abundances of diatom growth forms found on the surface of epiphytic sessile microfauna in different months (based on all replicates collected in Terra Nova Bay).

**Fig 6:**
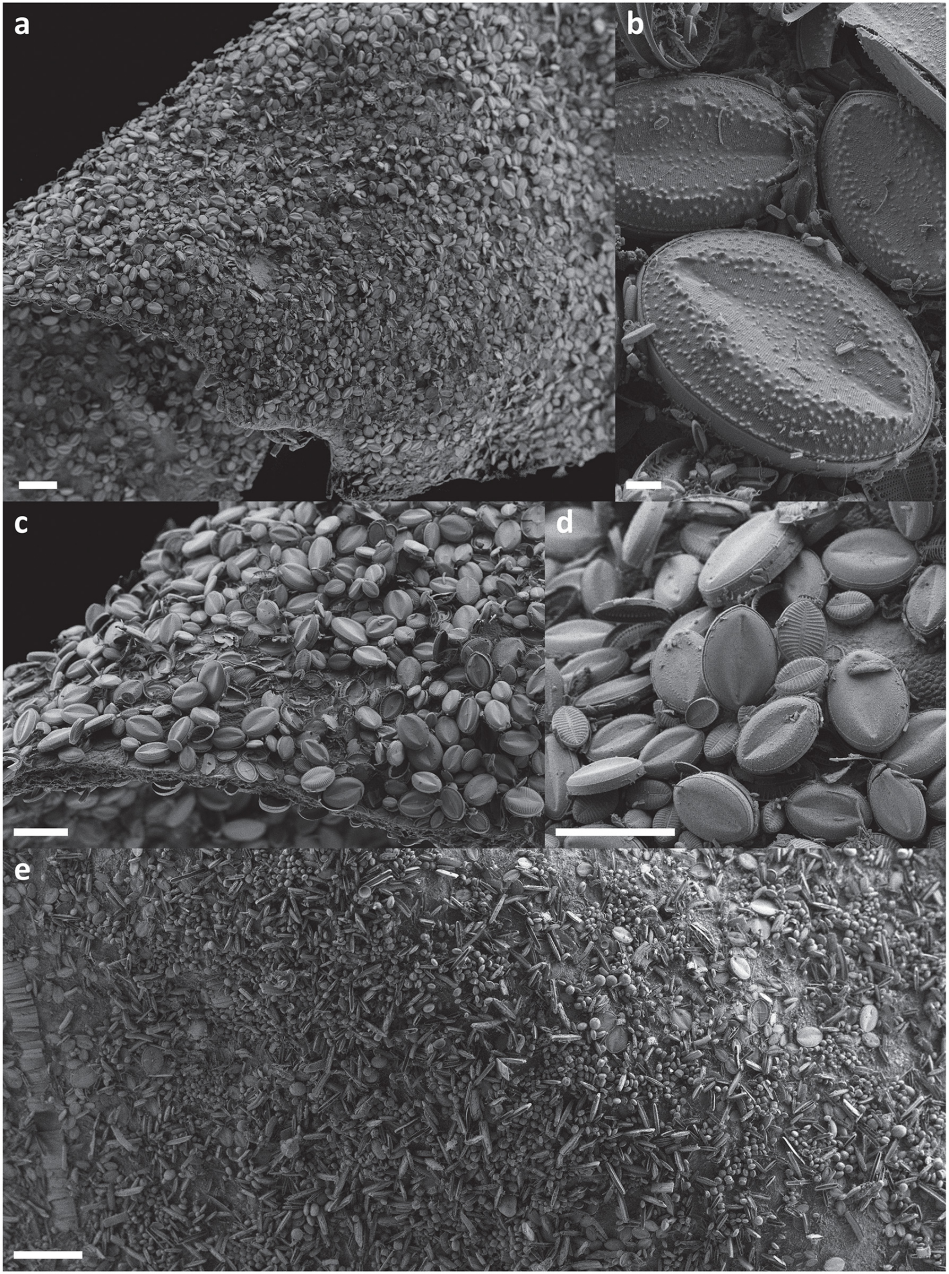
Scanning electron micrographs of epiphytic diatoms from the Ross Sea. **a.-d.** *Iridaea cordata* covered by dominating *Cocconeis antiqua* (Adélie Cove, Terra Nova Bay). **e.** surface of *Phyllophora antarctica* (Cape Evans, McMurdo Sound). Scale bars: a. & e. = 200 μm; b. = 10 μm; c. & d. = 100 μm.

Seasonal change was evident in case of both epiphytic and epizooic communities (Figs 4 and 5, S1 and S2 Figs). Epiphytic communities developed in December contained a high number of adnate forms (e.g. *Achnanthes vicentii*, *Amphora* spp., *Cocconeis* spp; Fig 6a–6d) and low number of planktonic ones (Fig 4, S1 Fig). In January, a substantial change was observed: the number of adnate forms remained almost constant while other groups doubled (erect), tripled (plocon), or increased their number by seven- (motile) or eleven-fold (planktonic; Fig 4), which affected significantly the community growth form structure (S1 Fig). Planktonic species such as *Fragilariopsis curta* and *F*. *nana* contributed up to 14.2 and 34.2% (Table 2), respectively, of the total diatom community developed on the seaweed thallus surface in January. In February, motile diatom (*Navicula* spp., *Nitzschia* spp.) numbers continued to grow. A slight increase was also noted in the case of adnate forms, while abundances of all other diatom groups decreased (Fig 4). Although adnate forms were the most important group in December, motile diatoms gained dominance as the summer advanced (S1 Fig). A similar seasonal shift in growth form structure was observed for epizooic communities, which, however, contained a much lower percentage of adnate forms and higher of erect ones than found in epiphytic samples (Fig 5, S2 Fig).

### Sampling site

**Fig 7:**
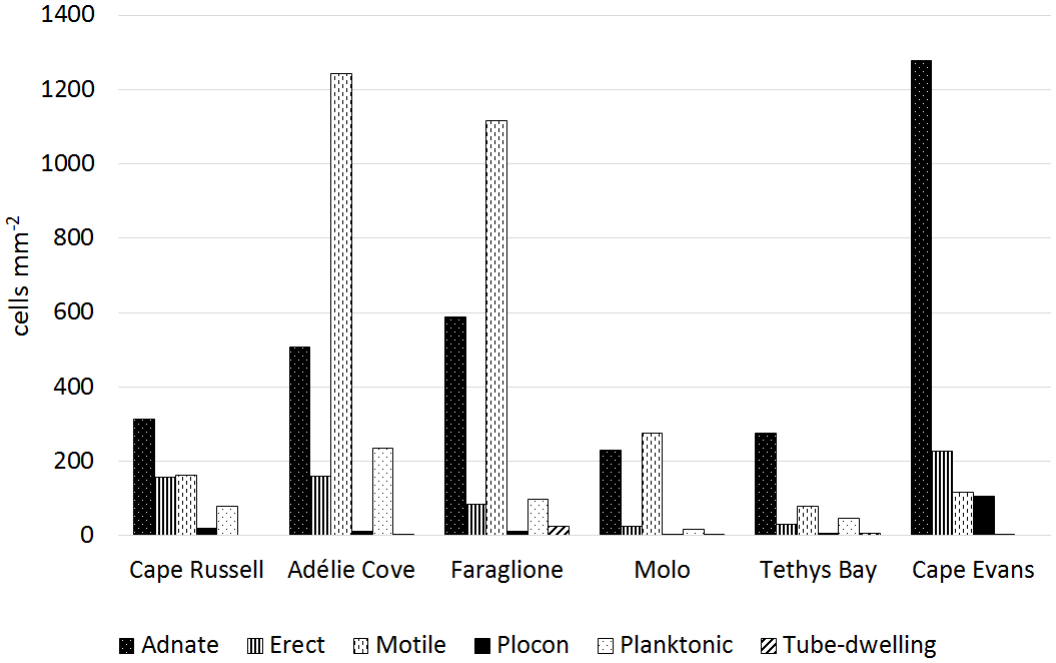
Average abundances of diatom growth forms found on macroalgal surface at selected sampling sites.

Differences in growth form structure among sampling sites are presented in Fig 7 (see also S3 Fig). Adnate forms dominated at Cape Russell, Tethys Bay, and Cape Evans, while the other three stations (Adélie Cove, Faraglione, Molo) were characterized by higher percentage of motile forms. The highest average total diatom number was found at Adélie Cove (2155 cells mm^-2^) and Faraglione (1925 cells mm^-2^), and the lowest at Tethys Bay (444 cells mm^-2^; Fig 7). It must be noted, however, that unequal number of samples collected at each of the sampling sites may have had some influence on the results obtained, as factors such as sampling season and depth also affected the communities.

### Depth

**Fig 8:**
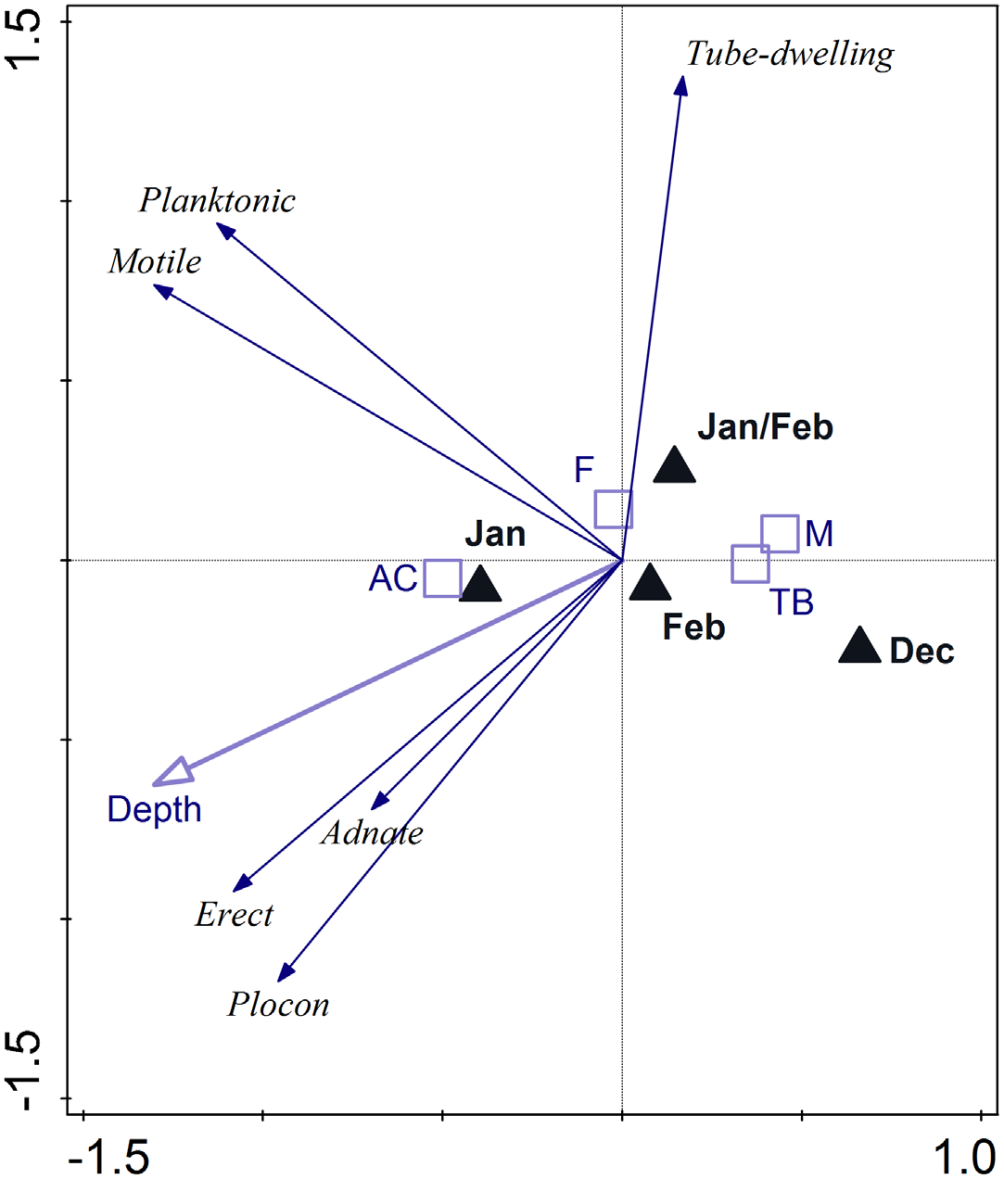
Biplot diagram from RDA (redundancy analysis) visualizing the effect of depth on different diatom growth forms. Supplemental environmental variables (sampling site, season), are added. Analyses is based on 166 subsamples. Due to the relatively low number of samples, results for Cape Evans and Cape Russell sampling sites are not shown. Score scaling is focused on growth form scores (standardized). Eigenvalues: 0.1225, 0.0309, 0.0092; p = 0.005. Adjusted explained variation = 10.3%. CR—Cape Russell, AD—Adélie Cove, F—Faraglione, M—Molo, TB—Tethys Bay.

Fig 8 visualizes the effect of depth on different diatom growth forms. Supplemental environmental variables (sampling site, season) were added as dummy variables. The adjusted explained variation accounted for 10.3% and the Monte Carlo permutation test (p < 0.001) confirmed that the observed effect was significant. Erect and adnate diatoms as well as plocon appeared to be positively correlated with depth, while tube-dwelling forms exhibited a negative correlation. For motile and planktonic species, the correlation was weak or almost non-existent. December was clearly indicated as the season in which the conditions were the least favourable for diatom community development. All of the diatom groups responded positively to the conditions of Adèlie Cove and/or Faraglione.

### Epiphytic microfauna

**Fig 9:**
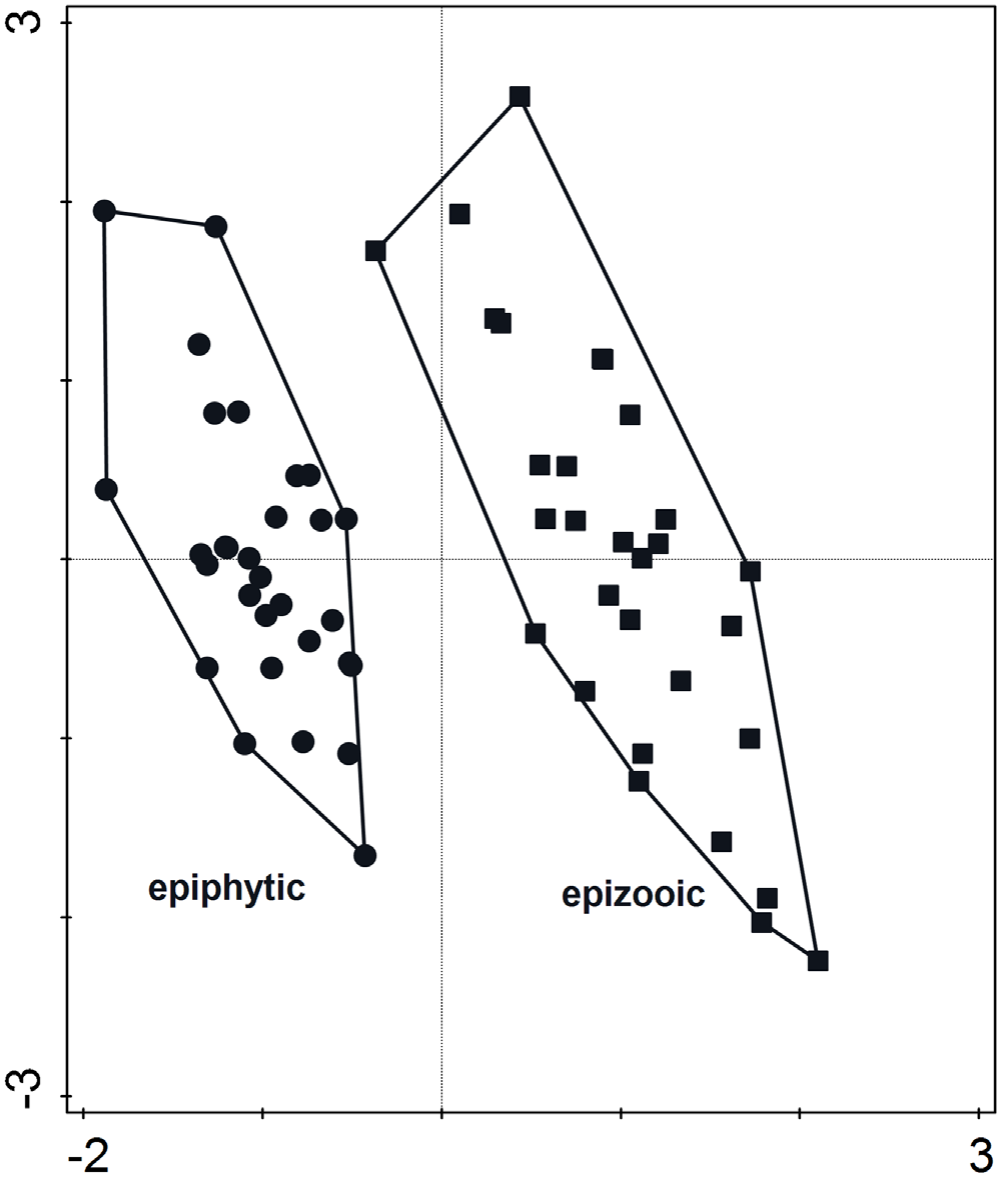
*Phyllophora* and *Plocamium* samples classified into two groups according to the host organisms: macroalga (epiphytic) or microfauna (epizooic). The polygons are plotted in the space of the first RDA axes. Score scaling is focused on diatom taxa scores (standardized). Eigenvalues: 0.1967, 0.0994, 0.0706; p = 0.0002. Adjusted explained variation = 27.0%.

**Fig 10:**
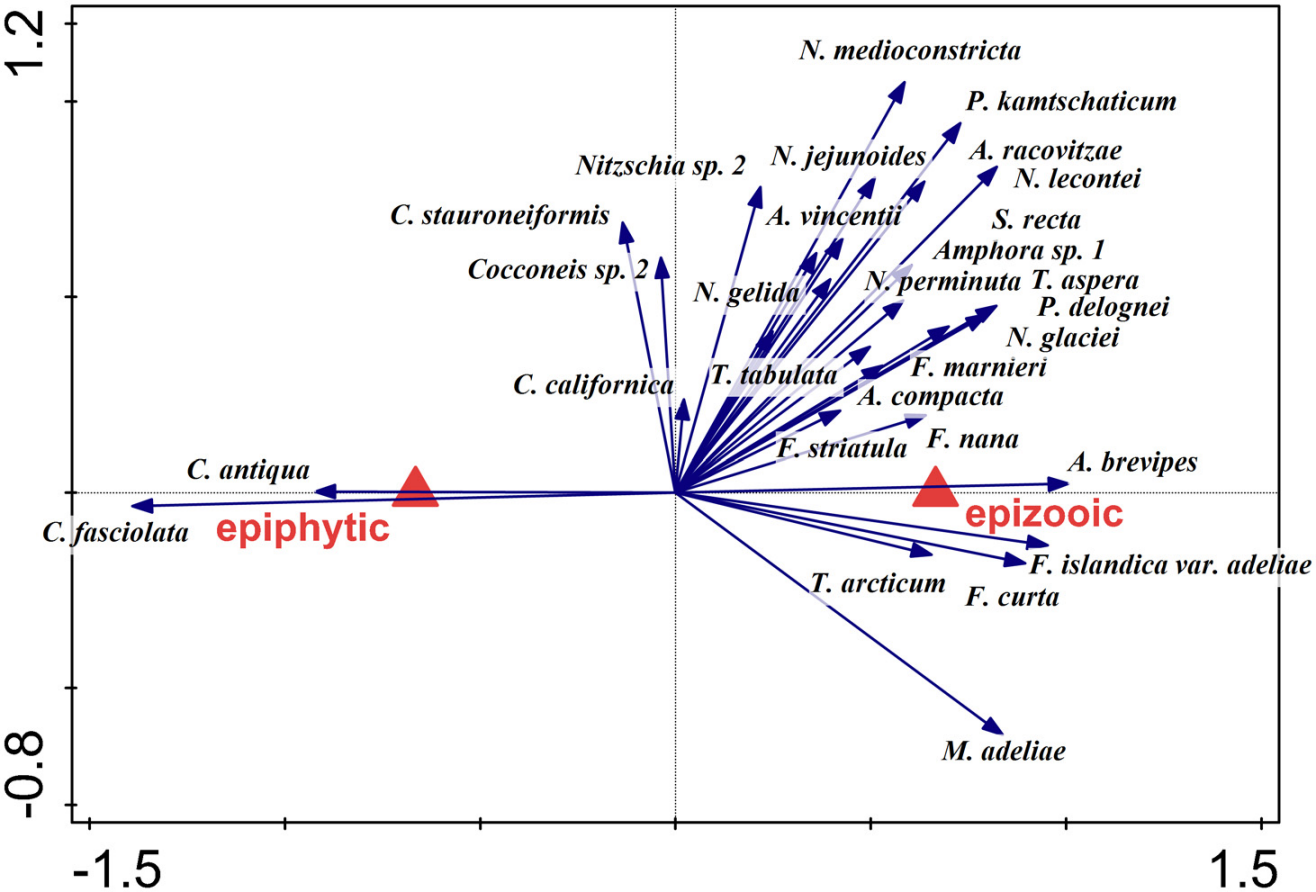
Biplot diagram from a partial redundancy analysis (RDA) summarizing the variation in diatom species composition explained by the host organism (macroalga or microfauna), after removal of the effect of sampling site, season and depth of sampling. Score scaling is focused on diatom taxa scores (standardized). Abundance data are log-transformed. Eigenvalues: 0.1967, 0.0994, 0.0706; p = 0.0002. Adjusted explained variation = 27.0%.

**Fig 11:**
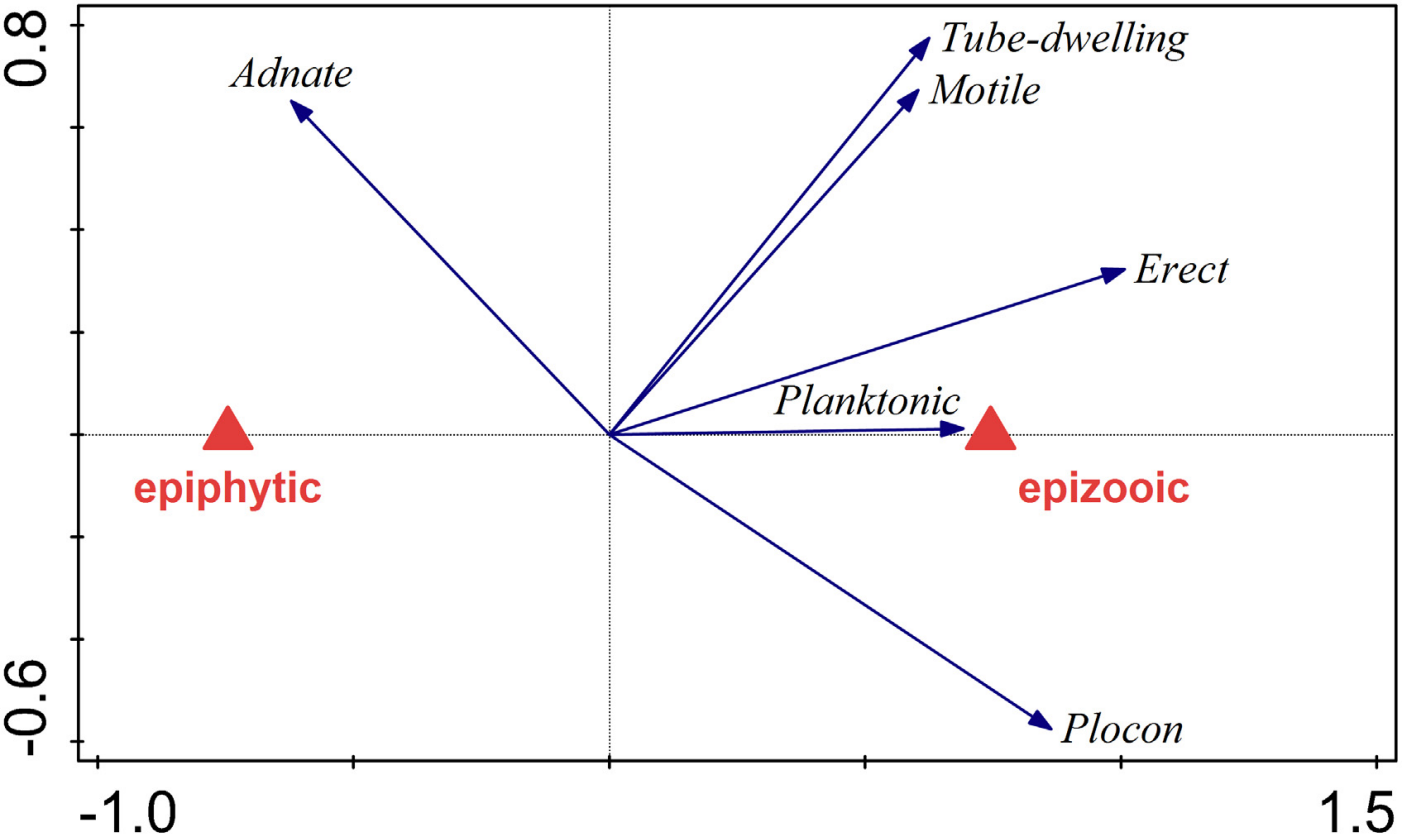
Biplot diagram from a partial redundancy analysis (RDA) summarizing the variation in diatom growth form explained by the host organism effect (macroalga or microfauna), after removal of the effect of sampling site, season and depth of sampling. Score scaling is focused on diatom growth form scores (standardized). Abundance data are log-transformed. Eigenvalues: 0.3084, 0.1085, 0.0969; p = 0.0002. Adjusted explained variation = 47.1%.

**Fig 12:**
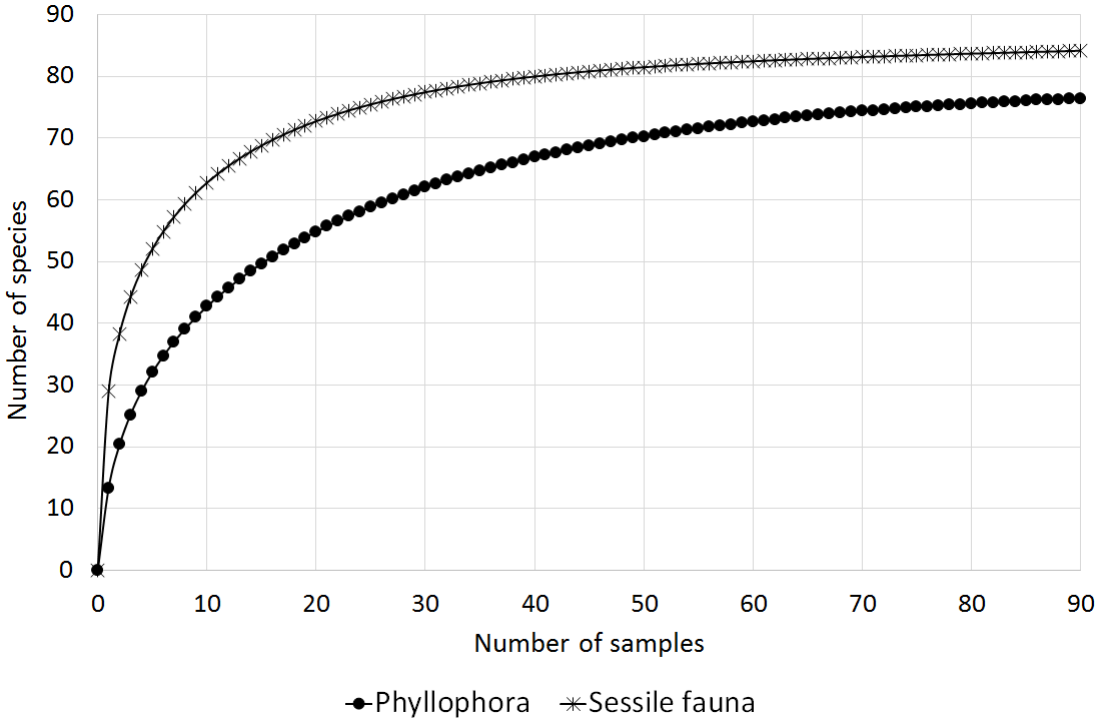
Extrapolation (plain lines) of rarefaction curves (lines with symbols) based on samples of *Phyllophora* and its associated sessile fauna from locations where both were found (Cape Russell, Adelie Cove, Faraglione, Molo, Tethys Bay).

**Fig 13:**
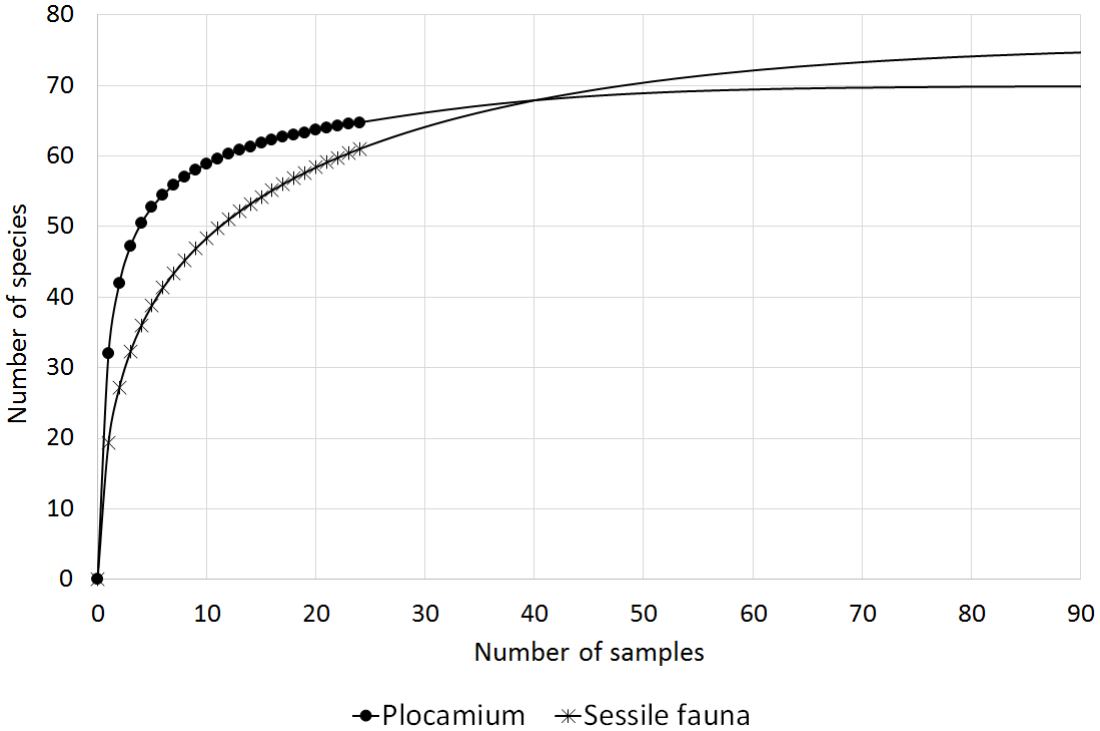
Extrapolation (plain lines) of rarefaction curves (lines with symbols) based on samples of *Plocamium* and its associated sessile fauna.

A partial RDA summarized the variation in diatom species composition explained by the host organism (macroalga or microfauna), after removal of the effect of sampling site, season and depth of sampling. Diatoms found in *Phyllophora* and *Plocamium* samples were classified into two groups according to the host organisms (as indicated above). The polygons in Fig 9 were plotted in the space of the first RDA axes. Score scaling was focused on diatom taxa scores (standardized). The explained adjusted variation accounted for 27% of the total variance in diatom compositional data. According to the Monte Carlo permutation test (p < 0.001) this effect was significant. Epiphytic and epizooic samples formed two distinct clusters, indicating a substantial difference between the communities (Fig 9). Only *Cocconeis antiqua* and *C*. *fasciolata* preferred clearly the specific ecological conditions created by host macroalga. Other *Cocconeis* species gave either slightly positive (*C*. *stauroneiformis*) or slightly negative (*C*. *californica*) responses to macroalgal substrate, while all other taxa preferred a micro-faunal surface (Fig 10). A partial RDA performed on growth form abundance data confirmed these observations and revealed even stronger influence of host organism type on diatom communities (adjusted explained variation = 47%, p < 0.001; Fig 11). Planktonic species showed very high affinity for microfaunal substrate, while adnate diatoms were the only group that preferred macroalgal over microfaunal substrate. The estimated species richness appeared to be higher for epizooic than epiphytic samples in the case of communities associated with both *Phyllophora* and *Plocamium* (Figs 12 and 13). Moreover, epizooic communities were usually characterized by significantly higher total diatom number than epiphytic ones (S4 and S5 Figs, Figs 4 and 5).

## Discussion

The number of diatom taxa found in this synthesis (109) is high in comparison with earlier studies of Antarctic epiphytes [32, 56, 63] and epiphytic diatoms in general [64, 65, 66, 67, 68]. Al-Handal and Wulff [56] reported 50 diatom taxa epiphytic on 19 different host species, including red, brown, and green macroalgae from Potter Cove (King George Island, South Shetland Islands) sampled between October and December 2003. Sutherland [63] observed 31 diatom species associated with *Phyllophora antarctica* in material collected from Cape Evans in November 2001. Thomas and Jiang [32] examined surface-associated diatoms near Davis Station (Prydz Bay) in a year-round study including 15 macroalgal taxa, but only identified nine diatom taxa. As reported by Majewska *et al*. [29, 30, 31], epiphytic diatom communities from the Southern Ocean may differ substantially between geographic locations and seasons. Nevertheless, we believe that at least some of the differences in the number of diatom taxa detected were underlain by the differences in methodology used. It has been observed that “traditional” methods of sampling and material processing may strongly influence the results obtained [69, 70, 71, 72]. Procedures involving corrosive substances may damage or completely dissolve weakly silicified diatom frustules, whereas taxa that do not cluster and settle to the bottom of the tube or beaker during the cleaning procedure may easily be overlooked and removed when rinsed [29]. Moreover, sampling effort clearly has a strong influence on the probability of species detection. Previous studies have indicated that careful analysis of 10 replicates of a total surface area of 20 mm^2^ is sufficient to allow detection of 85–95% of co-existing epiphytic diatom taxa [28], when used in concert with appropriate preparation and observation methods.

### Host macroalgal species and morphology

**Fig 14:**
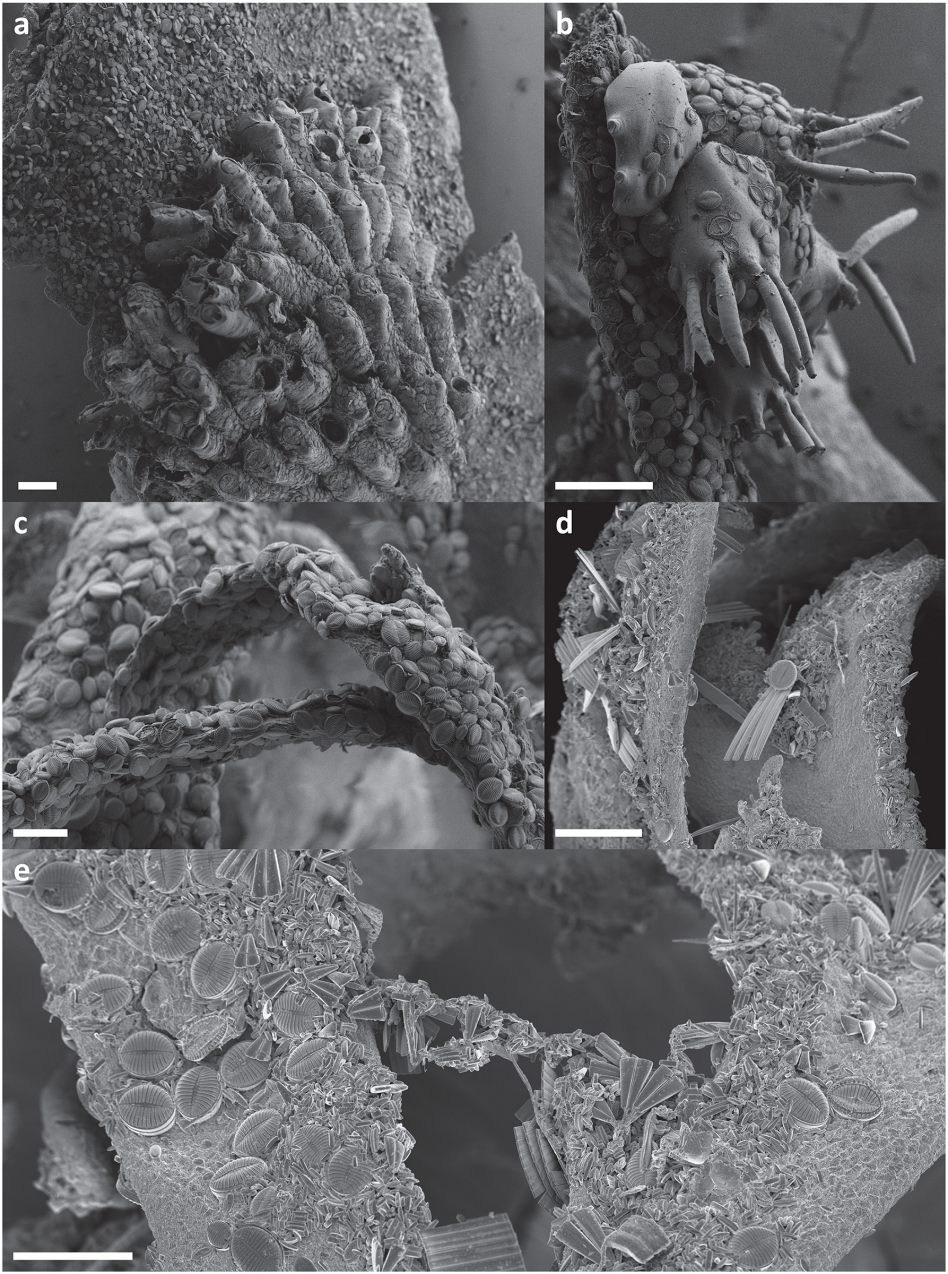
Scanning electron micrographs of epiphytic diatoms from Terra Nova Bay. **a. & b**. examples of epiphytic sessile fauna. **c.–e.** diatoms on *Plocamium cartilagineum*. Scale bars: a. & b. = 200 μm; c.-e. = 100 μm.

Debate continues over the importance of the host macroalgal species for associated microalgal communities. Several studies have suggested that the macroalgal basibiont influences epiphytic communities to a significant degree and indicate that at least some interactions between macroalgae and epiphytes may be species-specific [67, 73, 74]. Investigations worldwide as well as some studies conducted in the polar regions give contrasting results and interpretations [29, 30, 56, 75, 76]. Thomas and Jiang [32], working in the vicinity of Davis Station (Vestfold Hills, Princess Elizabeth Land), reported that epiphytic diatom community composition depended strongly on macroalgal morphology, with filamentous parts of the macroalgae hosting a different (and usually more diverse) diatom community than broad, folious blades. Our findings are consistent with this observation, with the diatom flora associated with filamentous *Plocamium cartilagineum* differing most clearly from those growing on the other two flat-bladed seaweed species (Figs 6 and 14) [29, 30]. The specific branching pattern of alternating groups of branchlets in pectinate series that is characteristic of *Plocamium* appeared to create an appropriate and sheltered environment for the attachment of many erect (*Fragilaria* spp., *Pseudogomphonema kamtschaticum*), chain-forming (*Grammatophora* spp.), or loosely attached (*Paralia sol*, *Trigonium arcticum*) diatoms (Fig 14c–14e). In the case of diatom communities associated with *Iridaea cordata* and *Phyllophora antacrtica*, due to the topographical similarity of substrate provided by relatively broad macroalgal blades, significant differences in diatom growth form and structure were not found. However, the diatom communities hosted by these latter two macroalgae differed greatly in the dominance of particular diatom species. Most strikingly, of the two dominant *Cocconeis* species, *C*. *antiqua* was always the most numerous among adnate diatoms on *Iridaea*, while *C*. *fasciolata* covered all *Phyllophora* samples densely [29, 30]. Both *Iridaea* and *Phyllophora* belong to Rhodophyta, both possess flat-bladed thalli and often occurred at the same sampling site. Host selection by the different diatom species is clearly effective. Dominating *Cocconeis* species have the same growth form and occupy apparently similar niches. While the homogenous microhabitat provided by macroalgal thalli might appear to favour competitive ability amongst coexisting species, preferences for a host macroalga would diminish the strength of interactions between diatom taxa and ensure their long-term ecological success [77, 78].

### Seasonality and ice

Although the data obtained focus on diatom communities developing during the three summer months, a strong seasonal influence was apparent. This is consistent with well-established understanding of Southern Ocean primary productivity, which is characterised by extreme seasonality [79, 80]. In the shallow coastal zone, sea ice has a very strong influence on coastal communities, altering local environmental characteristics and giving rise to distinct microbial assemblages [81, 82, 83]. Several marine microalgal species use different types of sea ice as temporary winter habitat [84, 85, 86]. During the warmer summer season, diatoms melt out of the ice seeding the water column [81] and other available habitats, including seaweed surfaces [28, 29, 30]. Studies have shown that in various Antarctic locations and times of the year, unique ice conditions support blooms of different diatom taxa [80, 87, 88, 89, 90, 91]. Several studies have reported blooms of *F*. *curta* and *F*. *cylindrus* seeded by melt-out of either sea ice or the marginal ice-edge [84, 86, 92]. Leventer and Dunbar [93] suggested that more diverse summer diatom assemblages are typical of regions influenced strongly by wind, while Arrigo and McClain [94] and Cunningham and Leventer [81] reported that summer diatom-dominated blooms in Terra Nova Bay are directly linked to the ice edge recession.

Macroalgal thalli collected from Terra Nova Bay in December and the first week of January, as well as those collected in January from Cape Evans (i.e. samples collected from under the ice or directly after ice break-up forced by wind and wave action) contained a high number of adnate forms (e.g. *Achnanthes vicentii*, *Amphora* spp., *Cocconeis* spp; Fig 6a–6d). Epontic, loosely-attached species, sometimes described as “plocon” [95], were present (e.g. *Melosira* spp., *Trigonium arcticum*), but planktonic species were almost absent. However, as the summer season advanced, species with other growth forms increased in abundance relative to sessile adnate diatoms. In January, a high number of planktonic and ice-associated diatoms such as *Fragilariopsis curta* and *F*. *nana* were present in samples collected in Terra Nova Bay [28, 29, 30]. Other *Fragilariopsis* species (*F*. *cylindrus*, *F*. *kergulensis*, *F*. *obliquecostata*, *F*. *rhombica*, *F*. *ritscheri*, *F*. *sublinearis*) were also present but in much lower numbers. Many samples contained *Chaetoceros* spp. resting spores. Several studies have indicated that *Thalassiossira antarctica* is another diatom closely associated with the coastal sea ice [89, 96, 97], but its environmental role is not fully understood [81]. In epiphytic samples, *T*. *antarctica* appeared only sporadically and was poorly represented, which may support a previous observation that this species rarely blooms in the coastal zone of Terra Nova Bay [81]. Simultaneously, numbers of small motile *Navicula* spp. and *Nitzschia* spp. began to increase, suggesting that these taxa also belong to the ice-associated group and are released into the water column as the ice melts. The contribution of ice-seeded *Fragilariopsis* species to the total diatom number decreased as the season progressed further (mid-February), but numbers of *Navicula* spp. and *Nitzschia* spp. increased rapidly and continued to increase until the end of February when the observations were terminated [29, 30].

Adnate taxa are often described as early colonizers that initiate algal succession on newly available substrates [98, 99]. It has been suggested that horizontally-growing epibiotic diatoms, staying in close contact with the host organism tissues, may benefit from the exchange of biogenic substances with the basibiont [100, 101]. Furthermore, adnate forms are highly resistant to both physical disturbance and grazing by various unspecialised herbivores [102]. Many function well in low-light conditions, for instance in relatively deep water, under ice-cover, or overshadowed by apically attached, stalked, or loosely-associated diatoms or settled planktonic forms [102, 101]. In our studies, after ice break-up the average number of adnate forms did not decrease substantially, but small motile diatoms achieved dominance when the *Fragilariopsis* spp. bloom began to decline [29, 30]. In Terra Nova Bay summer diatom blooms and high uptake of biogenic substances may result in significant nutrient depletion [103, 104, 105]. In such conditions, small-celled diatoms, as opposed to large-celled *Cocconeis* (*C*. *antiqua*, *C*. *fasciolata*) and bulky *Amphora* species, have an advantage because of their high surface area to volume ratio, which allows them to more easily satisfy their nutrient demand [106]. Sunda and Huntsman [107] suggested that small algae have lower iron requirement than larger taxa, while their growth rates are much higher. In addition, highly motile *Navicula* and *Nitzschia* species are able to migrate in response to various environmental factors, including nutrient gradients and irradiance [108]. They are thus excellent colonizers or re-colonizers of newly-exposed surfaces, and can dominate in habitats where low levels of nutrients inhibit or moderate the growth of less tolerant diatoms. Their populations can recover rapidly from disturbances such as grazing or mechanical removal by physical forces [12, 100, 109] in comparison with large-celled species.

A gradual decrease of *Fragilariopsis* spp. percentage contribution in epiphytic samples from Terra Nova Bay [29, 30] may indicate that diatoms belonging to this genus are less resistant to the sudden nutrient depletion that occurs after the phytoplankton summer peak than are *Navicula* or *Nitzschia* spp, or that these araphid diatoms are not competitive in the new (epiphytic) habitat. Ice-associated diatoms such as *Amphiprora* spp., *Entomoneis* spp., *Nitzschia stellata* and other biraphid, motile taxa are truly benthic, being dependent on the sea ice as an “inverted benthos”, and do not survive and thrive once released into the water column, unlike many *Fragilariopsis* spp. (Amy Leventer, personal communication). Therefore, biraphid diatoms may find adequate habitat on solid surfaces such as macroalgal thalli, while *Fragilariopsis* spp., able to persist in the water column, require lower competitive abilities. In other words, there may be less pressure on *Fragilariopsis* spp. to colonize macroalgae rapidly, as the availability of solid surfaces is not a limiting factor.

The exchange of substrate between ice-associated diatoms and epiphytic forms has been suggested to have only marginal significance [56, 63, 110]. However, our data demonstrate that this depends largely on the season and ice conditions in the investigated area [28, 29, 30, 31]. Benthic or epontic forms constitute a substantial proportion of the diatoms that are able to survive in brine pockets in the winter ice. In contrast with oceanic planktonic taxa, benthic diatoms evolved in highly variable shallow water conditions and are likely to cope better with the high osmotic pressure fluctuations typical of ice-occluded microhabitats [111, 112]. Several studies have indicated that sea ice algae serve as an inoculum for the phytoplankton blooms associated with ice melt in the spring [111, 113, 114]. We suggest that a similar scenario is applicable to many of the ice-related forms of benthic origin, whose ice-associated mode of existence represents a stage of their natural life cycle.

Similar patterns were apparent in samples collected across 11 different years between 1989/1990 and 2012/13. Thus, we suggest that the seasonal effect is stronger than natural variability, and that the changes described are associated directly with local ice conditions [28, 29, 30, 31]. Cunningham and Leventer [81] stressed the importance of the Terra Nova Bay polynya in establishing the algal communities in this zone. However, in terms of both species composition and growth form structure, diatom communities from Tethys Bay observed in the current study collected immediately after ice break-up were more similar to those collected from under the ice at remote Cape Evans than to samples from nearby stations located along the coast of Terra Nova Bay. As the Tethys Bay samples were collected ca. 12-48h after the ice break-up, the diatom communities observed in those samples were still similar to the under-ice, “winter” assemblages and differed significantly from the nearby stations (Molo, Faraglione, Adélie Cove) where the ice broke up several weeks earlier. This supports the hypothesis that ice cover (and ice-associated drivers in general) has a profound influence on epiphytic diatoms and is one of the most important controls affecting polar marine microalgal communities.

### Sampling location

During the survey, amongst the six sampling sites located within Terra Nova Bay and at Cape Evans, the highest values of total diatom abundance were obtained in samples collected in the vicinity of Adélie Cove and Faraglione (> 8000 cells mm^-2^; Majewska *et al*. 2013b). The nearby penguin rookery and associated large influx of nutrients into the adjacent waters was proposed as a major factor influencing diatom number in this area [29,30]. Adélie Cove is a small, rather deep V-shaped bay, separated from the open sea by a ca. 15 m deep sill [115]. Povero *et al*. [115] described complex interactions among physical, chemical, and biological processes that influence local benthic and pelagic habitats, sustaining a particularly rich (in terms of both species diversity and biomass) benthic fauna. Adélie Cove and Faraglione are under strong terrestrial influences, including not only the nearby Adélie penguin (*Pygoscelis adeliae*) rookery, but also intense katabatic winds which strongly influence the local water mass circulation. The underwater sill and other morphological features of the coast favour nutrient entrapment within the cove [115]. The amount of organic matter found in sediments at 50 m depth was 3–4 times higher than the quantities measured at other sampling stations within the bay [115, 116]. Intense *Phaeocystis* blooms, followed by summer (January-February) diatom blooms are observed regularly in the vicinity of the cove [115] (Maria Cristina Gambi, personal communication; Majewska, personal observation) and the high organic matter content is thought to support development of particularly abundant communities of detritus-feeding and suspension-feeding benthic organisms [115, 117]. However, as noted by Povero *et al*. [115], due to wind action and water circulation pattern the influence of the penguin colony may be even stronger in the vicinity of Faraglione. The observations of Andreoli *et al*. [118] of unusually high densities of planktonic microalgae in this area, may support these inferences.

The least abundant epiphytic diatom communities (from 21 to 1312 cells mm^-2^) [28, 30] were found in Tethys Bay, a station characterised by particularly unstable summer conditions (strong winds, ice break-up during the study period) and potential mechanical disturbance and damage. Comparing to other sampling sites located within Terra Nova Bay, the macroalgal fauna found in Tethys Bay was relatively poor, which was probably directly related to the longer period of ice cover.

Epiphytic diatom abundances at Cape Evans, located near the southern global limit of macroalgal growth [119], did not differ significantly from those observed at Terra Nova Bay. This suggests that relatively thin (up to 1 m) ice cover and low water temperatures (ca. -1.4°C) do not significantly affect diatom growth rates and biomass. Similar conclusions have also been drawn by Thomas and Jiang [32], who observed that the standing crop of epiphytes from the vicinity of Davis station was high throughout both summer and winter seasons. Miller and Pearse [9] suggested that low temperatures depress respiration rates and thus favour growth and survival in low light conditions.

### Depth and light conditions

Data obtained in the current study indicated that the depth of sampling was of secondary importance for diatom community development [29, 30]. In terms of diatom growth form structure, there was a general trend towards an increasing number of erect forms in deeper waters, presumably owing to the poorer light conditions present at deeper stations. In addition, tube-dwelling diatoms seemed to prefer the shallowest sites (2–5 m). The deepest sampling station, however, did not exceed 25 m and clearly conditions will differ in deeper waters. Sutherland [63] noted a gradual decrease in diatom diversity at Cape Evans as the depth below the sea ice increased, and we suggest that depth can be an important factor limiting species distribution when the incident photosynthetically active radiation (PAR) that penetrates through the water column is just above the lowest values necessary for algal growth. The availability of PAR is a result of complex interactions among factors including water turbidity, suspended particles (organic and mineral), meteorological conditions (clouds), ice cover, substratum slope, or overshadowing by macroalgal beds [120, 121, 122, 123]. An erect position may be an adaptation to conditions where only a small portion of solar radiation is transmitted to the benthic or epiphytic habitats [122]. This may explain the relatively high number of erect diatoms found at Adélie Cove (despite high amounts of suspended particles and dense macroalgal beds) and Cape Evans (ice cover). The formation of mucilaginous tubes, in turn, may provide protection from desiccation, osmotic shock or intense light radiation in upper water layer [124, 125].

### Water currents

A complex, vertical structure of diatom communities may reflect other characteristics of the occupied habitat. As opposed to tightly-attached adnate diatoms, both erect and tube-dwelling diatoms are highly susceptible to physical disturbances such as current-induced shear forces [126]. Therefore, independent of light conditions, loosely attached diatoms find more favourable conditions for their growth at sheltered sites where they are not exposed to strong currents, wind-generated waves or ice scouring. Usually such conditions are typical for sites covered by ice over several months [127]. Ryan *et al*. [110] described the influence of water movement on ice algae at Cape Evans and Cape Hallett, indicating that the currents were negligible at the former location. Moreover they reported that, due to the non-existent or very weak water currents at Cape Evans, ice-associated algae were not present in the water column. Our findings are consistent with these observations, the very weak water currents at Cape Evans prevented exchange between ice-associated and epiphytic communities, resulting in the formation of very distinct assemblages [28].

The specific impact of water currents on epiphytic sessile microfauna, which have a functional significance for epiphytic diatoms, is discussed below. In the case of benthic suspension and deposit feeders, flow regime plays a crucial role in their feeding success. Under low flow conditions, food sources are easily depleted. Therefore, weak water and particle movements may negatively affect growth and survival of sessile invertebrates [128, 129, 130].

### Associated epiphytic microfauna

In contrast with the epiphytic communities of Terra Nova Bay, various growth forms of diatoms at Cape Evans covered the entire macroalgal thalli evenly [28]. All samples from Terra Nova Bay were also colonised to some degree by sessile microfauna (Fig 14a and 14b), while the Cape Evans macroalgae were free of associated fauna [28]. The associated microfauna has a pronounced influence on the local epiphytic diatom distribution [28, 29, 30, 31]. Generally, more diverse diatom forms tended to cluster on the surface and in the vicinity of sessile microfauna. The number and type of diatoms depended most likely on the microfaunal species or its morphology (e.g. adnate and small motile forms preferred the surface of associated bryozoans, while erect, loosely attached, and large motile forms preferred the vicinity of epiphytic hydroids; Majewska, personal observations), but the presence of a faunal component almost always increased both diatom number and species diversity (S4 Fig) [29, 30]. Topographically uniform macroalgal blades provide a low number of available types of microhabitat, supporting a low number of evenly-distributed epiphytic diatoms. Epiphytic communities clustered in the vicinity of sessile microfauna exhibited a tendency towards increasing biodiversity and more complex community structure, and thus differed substantially from the neighbouring, strictly epiphytic communities. Sessile fauna with their specific morphology, surface roughness and texture offer a wide range of microniches, which attracts high number of surface-associated diatoms [131, 132], while small planktonic forms may become trapped in cavities and depressions of the invertebrate surface [29, 30]. Epiphytic suspension-feeding fauna can also generate micro-eddies and thus increase the rate of particle settling [129, 130, 133], which may explain the high number of small planktonic diatoms (e.g. *Fragilariopsis nana*) concentrated in the vicinity of sessile epiphytic fauna found in our studies of Ross Sea macroalgae [29, 30].

There may also be a trophic relationship between the sessile fauna and their associated diatoms. Planktonic grazers produce microscale nutrient patches that are locally important for pelagic communities [134] and we suggest that similar process may occur in surface-associated communities, and further that epiphytic microalgae may profit from nutrients excreted by associated invertebrates. Such a relationship has been indicated by McCormick and Stevenson [135], who suggested that direct excretion of nutrients by epiphytic snails may increase nutrient availability to associated microalgae. Furthermore, the surface of living sessile animals may provide a favourable substratum for epibiont growth as these areas are usually subjected to weaker grazing pressure [95, 100]. The lack of sessile suspension-feeding organisms at Cape Evans might have been caused by low particle flux (a direct consequence of the absence or very low biomass of phytoplanktonic organisms in the water column beneath the ice) and very weak currents, as mentioned above [129, 136]. The highly patchy distribution of epiphytic diatoms in Terra Nova Bay may also be linked with extensive, non-selective grazing by a wide range of herbivorous organisms [28]. The shallow waters of Terra Nova Bay are especially rich in benthic fauna, many of which may feed on benthic diatoms [115, 129, 137].

## Conclusions

Epiphytic diatom communities investigated at various depths at six sites located in Terra Nova Bay and McMurdo Sound (Ross Sea) proved to be rich, well developed and diverse in terms of both species composition and growth form structure. Generally, the epiphytic diatom flora overlapped that of the sea ice to a relatively small extent, but the contribution of both ice-associated and planktonic diatoms to the epiphytic communities was related strongly to the local ice conditions and increased with progression through the summer season. Spring ice melt or break-up and seeding by ice-associated diatoms are important in determining the composition of epiphytic communities in summer. Although ice cover strongly influenced epiphytic diatom community composition, it neither inhibited their growth nor limited significantly their abundance. A wide range of environmental factors can influence diatom communities, but their effects can only be considered in relation to broader aspects of ecosystem functioning. The host macroalga influenced the associated diatoms mainly through its morphology and surface texture and roughness, providing a point of attachment and shelter for host-adapted species. However, other interactions (e.g. trophic, chemical) cannot be excluded. In general, less uniform surfaces supported a higher number of epiphytes, and the presence of epiphytic sessile fauna also increased significantly the local biodiversity. Depth affected diatom community growth form structure, which was manifested especially by higher contributions of erect forms to the total diatom number at the deepest stations sampled in this study. Nevertheless, in the generally shallow ice-free marine habitats examined, where water transparency is relatively high, the influence of depth was of only secondary importance for epiphytic communities. Our findings highlight the need for further, long-term and spatially extensive investigations to gather the necessary information about individual species of benthic marine diatoms, in order to permit their use as valid environmental proxies.

## Supporting Information

S1 Fig Average percent contribution of diatom growth forms to the total number of diatoms found on the macroalgal surface in different months (based on all macroalgal replicates collected in Terra Nova Bay). (TIF)

S2 Fig Average percent contribution of diatom growth forms to the total number of diatoms found on the surface of epiphytic sessile microfauna in different months (based on all replicates collected in Terra Nova Bay). (TIF)

S3 Fig Average percent contribution of diatom growth forms to the total number of diatoms found on the macroalgal surface at selected sampling sites. (TIF)

S4 Fig Average growth form abundances of diatoms associated with *Phyllophora antarctica* and *Plocamium cartilagineum*. CR—Cape Russell, AD—Adélie Cove, F—Faraglione, M—Molo, TB—Tethys Bay, Dec—December, Jan—January, Feb—February.
(TIF)

S5 Fig Average growth form abundances of diatoms associated with sessile fauna epiphytic on *Phyllophora antarctica* and *Plocamium cartilagineum*. CR—Cape Russell, AD—Adélie Cove, F—Faraglione, M—Molo, TB—Tethys Bay, Dec—December, Jan—January, Feb—February.
(TIF)

S1 Table List of samples. (DOCX)

S2 Table Average abundance of diatoms associated with *Phyllophora antarctica* and *Plocamium cartilagineum*, and their contribution to the dissimilarity found between the groups. (DOCX)

S3 Table Average abundance of diatoms associated with *Phyllophora antarctica* and *Iridaea cordata*, and their contribution to the dissimilarity found between the groups. (DOCX)

S4 Table Average abundance of diatoms associated with *Plocamium cartilagineum* and *Iridaea cordata*, and their contribution to the dissimilarity found between the groups. (DOCX)
