# Supplementary figures and images for: Summer Epiphytic Diatoms from Terra Nova Bay and Cape Evans (Ross Sea, Antarctica) - A Synthesis and Final Conclusions
Source: PLoS One. 2016 Apr 14;11(4):e0153254. doi: 10.1371/journal.pone.0153254 (PMC4831778; doi:10.1371/journal.pone.0153254)

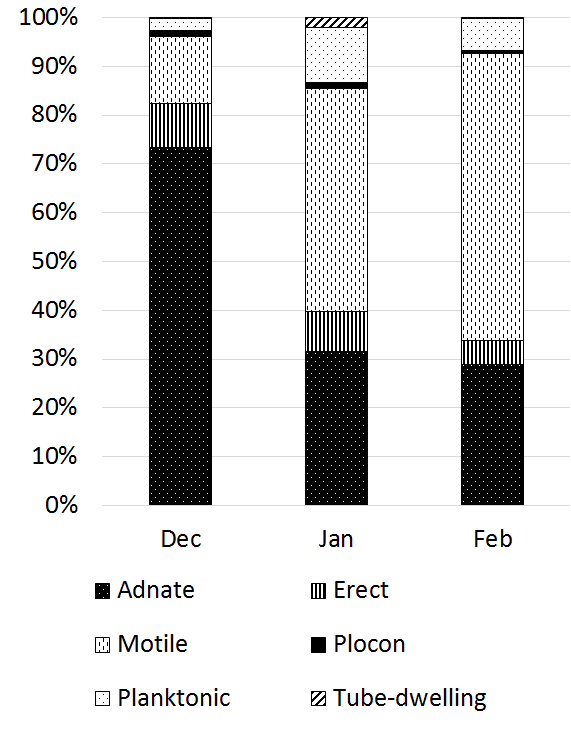

Supplement: S1 Fig — (TIF) [file pone.0153254.s001.tif]

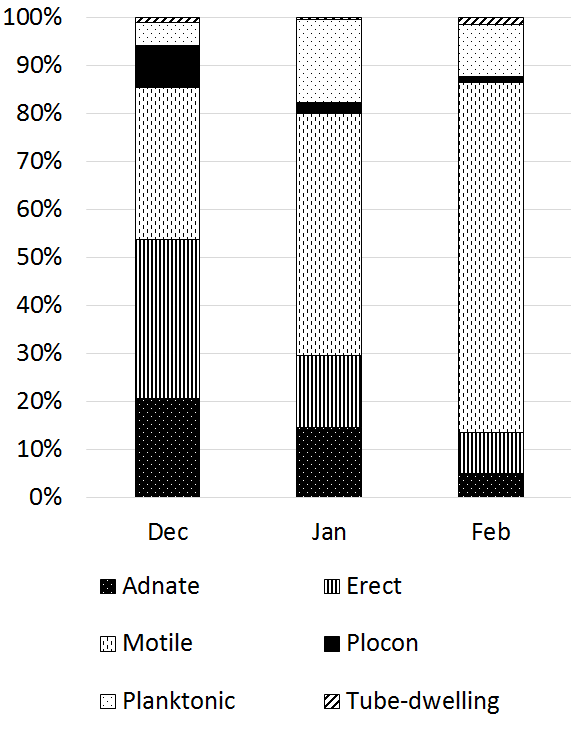

Supplement: S2 Fig — (TIF) [file pone.0153254.s002.tif]

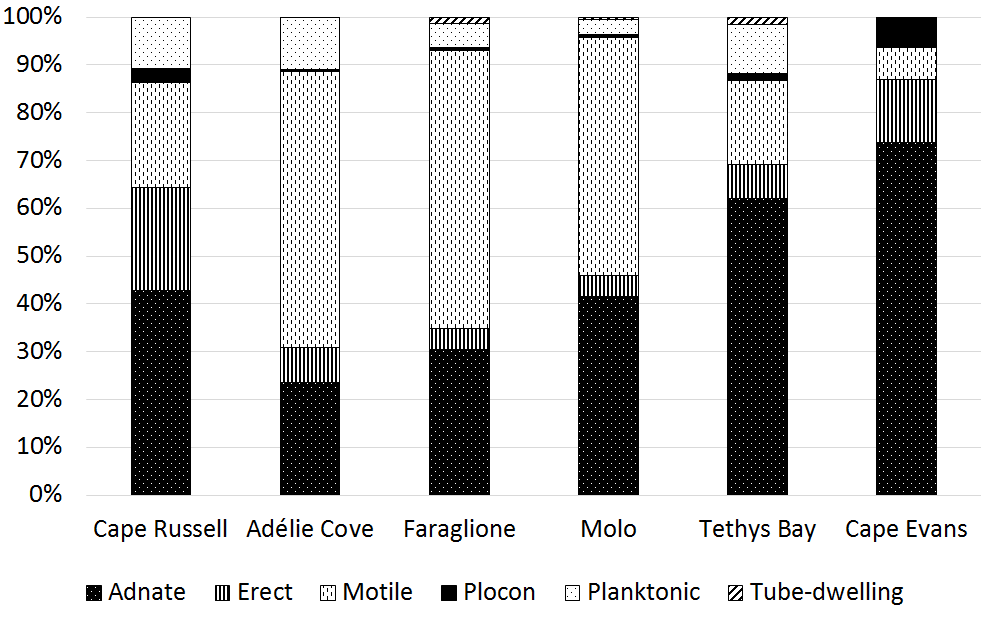

Supplement: S3 Fig — (TIF) [file pone.0153254.s003.tif]

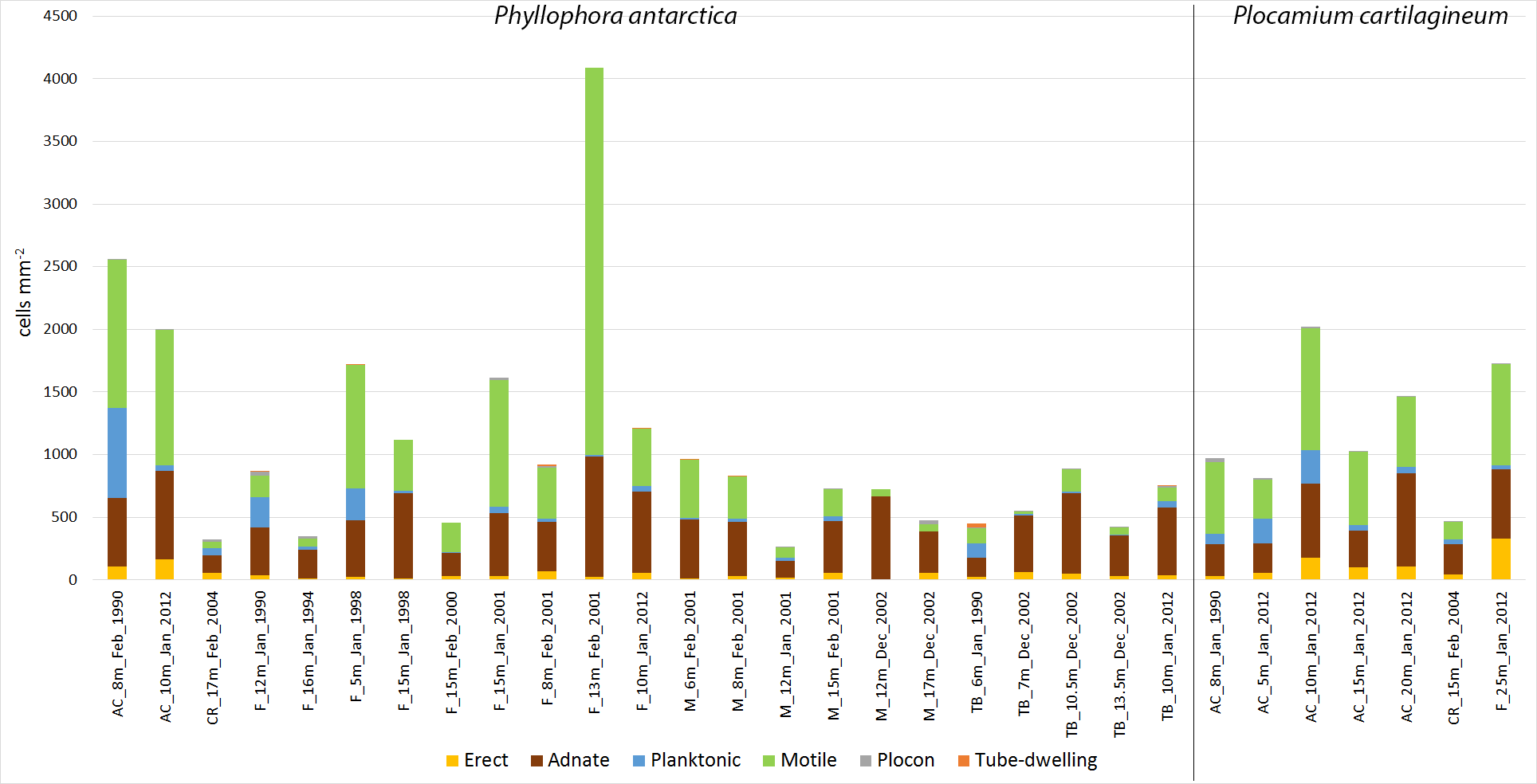

Supplement: S4 Fig — CR—Cape Russell, AD—Adélie Cove, F—Faraglione, M—Molo, TB—Tethys Bay, Dec—December, Jan—January, Feb—February. (TIF) [file pone.0153254.s004.tif]

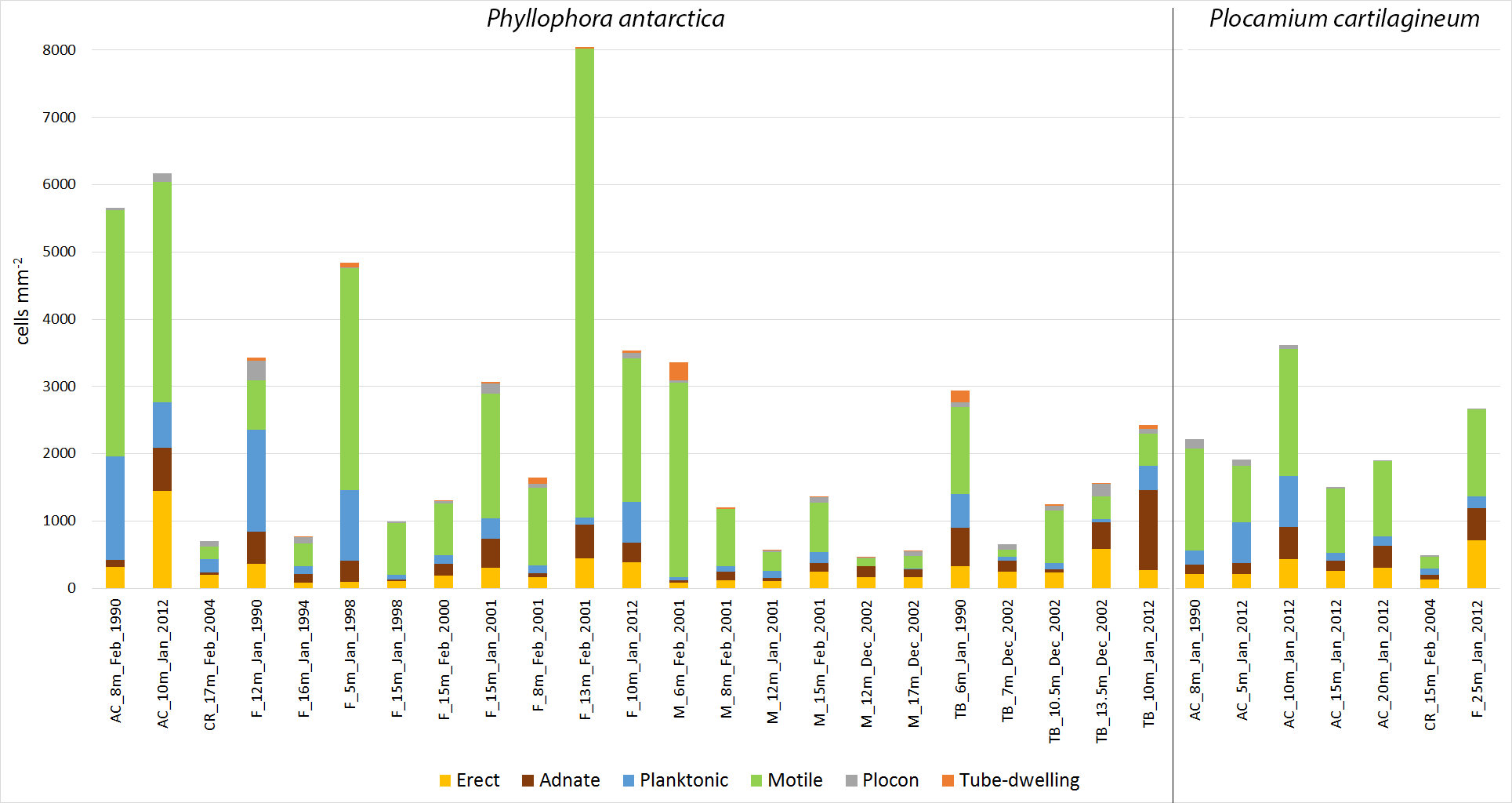

Supplement: S5 Fig — CR—Cape Russell, AD—Adélie Cove, F—Faraglione, M—Molo, TB—Tethys Bay, Dec—December, Jan—January, Feb—February. (TIF) [file pone.0153254.s005.tif]
