# Supplementary material for: Summer Epiphytic Diatoms from Terra Nova Bay and Cape Evans (Ross Sea, Antarctica) - A Synthesis and Final Conclusions
Source: PLoS One. 2016 Apr 14;11(4):e0153254. doi: 10.1371/journal.pone.0153254 (PMC4831778; doi:10.1371/journal.pone.0153254)
Supplement: S1 Table — (DOCX) [file pone.0153254.s006.docx]

**S1 Table** List of samples.

| Sampling station | Macroalgal host | Number of replicates | Depth (m) | Sampling date |
| --- | --- | --- | --- | --- |
| Cape Russell | *I. cordata* | 3 | 17 | Feb 2004 |
| Cape Russell | *P. antarctica* | 3 | 17 | Feb 2004 |
| Cape Russell | *P. cartilagineum* | 3 | 15 | Feb 2004 |
| Adélie Cove | *I. cordata* | 3 | 8 | Feb 1990 |
| Adélie Cove | *P. antarctica* | 3 | 8 | Feb 1990 |
| Adélie Cove | *P. cartilagineum* | 3 | 8 | Feb 1990 |
| Adélie Cove | *I. cordata* | 3 | 0.5 | Jan 1994 |
| Adélie Cove | *P. cartilagineum* | 3 | 0.5 | Jan 1994 |
| Adélie Cove | *P. antarctica* | 10 | 10 | Jan 2012 |
| Adélie Cove | *P. cartilagineum* | 3 | 5 | Jan 2012 |
| Adélie Cove | *P. cartilagineum* | 3 | 10 | Jan 2012 |
| Adélie Cove | *P. cartilagineum* | 3 | 15 | Jan 2012 |
| Adélie Cove | *P. cartilagineum* | 3 | 20 | Jan 2012 |
| Faraglione | *I. cordata* | 3 | 2 | Jan 1990 |
| Faraglione | *P. antarctica* | 3 | 12 | Jan 1990 |
| Faraglione | *P. antarctica* | 3 | 16 | Jan 1994 |
| Faraglione | *I. cordata* | 3 | 3 | Jan 1995 |
| Faraglione | *I. cordata* | 3 | 5 | Jan 1998 |
| Faraglione | *P. antarctica* | 3 | 5 | Jan 1998 |
| Faraglione | *P. antarctica* | 3 | 15 | Jan 1998 |
| Faraglione | *I. cordata* | 3 | 5 | Feb 2000 |
| Faraglione | *P. antarctica* | 3 | 15 | Feb 2000 |
| Faraglione | *I. cordata* | 3 | 6 | Feb 2001 |
| Faraglione | *I. cordata* | 3 | 8 | Feb 2001 |
| Faraglione | *P. antarctica* | 3 | 8 | Feb 2001 |
| Faraglione | *P. antarctica* | 3 | 13 | Feb 2001 |
| Faraglione | *P. antarctica* | 3 | 15 | Jan 2001 |
| Faraglione | *P. antarctica* | 10 | 10 | Jan 2012 |
| Faraglione | *P. cartilagineum* | 3 | 10 | Jan 2012 |
| Molo | *I. cordata* | 3 | 5 | Feb 1994 |
| Molo | *I. cordata* | 3 | 5 | Feb 1998 |
| Molo | *I. cordata* | 3 | 5 | Jan 2000 |
| Molo | *I. cordata* | 3 | 6 | Feb 2001 |
| Molo | *P. antarctica* | 3 | 6 | Feb 2001 |
| Molo | *P. antarctica* | 3 | 8 | Feb 2001 |
| Molo | *P. antarctica* | 3 | 12 | Jan 2001 |
| Molo | *P. antarctica* | 3 | 15 | Feb 2001 |
| Molo | *P. antarctica* | 3 | 12 | Dec 2002 |
| Molo | *P. antarctica* | 3 | 17 | Dec 2002 |
| Tethys Bay | *P. antarctica* | 3 | 6 | Jan 1990 |
| Tethys Bay | *I. cordata* | 3 | 7 | Dec 2002 |
| Tethys Bay | *P. antarctica* | 3 | 7 | Dec 2002 |
| Tethys Bay | *P. antarctica* | 3 | 10.5 | Dec 2002 |
| Tethys Bay | *P. antarctica* | 3 | 13.5 | Dec 2002 |
| Tethys Bay | *P. antarctica* | 10 | 10 | Jan 2012 |
| Cape Evans | *P. antarctica* | 10 | 10 | Jan 2011 |
