# Supplementary material for: Summer Epiphytic Diatoms from Terra Nova Bay and Cape Evans (Ross Sea, Antarctica) - A Synthesis and Final Conclusions
Source: PLoS One. 2016 Apr 14;11(4):e0153254. doi: 10.1371/journal.pone.0153254 (PMC4831778; doi:10.1371/journal.pone.0153254)
Supplement: S2 Table — (DOCX) [file pone.0153254.s007.docx]

**S2 Table** Average abundance of diatoms associated with *Phyllophora antarctica* and *Plocamium cartilagineum*, and their contribution to the dissimilarity found between the groups.

|  | Average abundance | | Average dissimilarity | Contribution (%) | Cumulated (%) |
| --- | --- | --- | --- | --- | --- |
|  | *Phyllophora* | *Plocamium* |  |  |  |
| *Navicula perminuta* | 20.19 | 25.57 | 6.28 | 11.71 | 11.71 |
| *Cocconeis fasciolata* | 18.08 | 13.19 | 3.08 | 5.57 | 17.46 |
| *Fragilariopsis nana* | 6.97 | 9.96 | 2.98 | 5.56 | 23.01 |
| *Achnanthes vicentii* | 8.3 | 11.49 | 2.96 | 5.52 | 28.54 |
| *Pseudogomphonema kamtschaticum* | 3.69 | 5.94 | 1.89 | 3.51 | 32.05 |
| *Tabularia tabulata* | 2.76 | 4.73 | 1.78 | 3.32 | 35.37 |
