# Supplementary material for: Summer Epiphytic Diatoms from Terra Nova Bay and Cape Evans (Ross Sea, Antarctica) - A Synthesis and Final Conclusions
Source: PLoS One. 2016 Apr 14;11(4):e0153254. doi: 10.1371/journal.pone.0153254 (PMC4831778; doi:10.1371/journal.pone.0153254)
Supplement: S3 Table — (DOCX) [file pone.0153254.s008.docx]

**S1 Table** List of samples.

| Sampling station | Macroalgal host | Number of replicates | Depth (m) | Sampling date |
| --- | --- | --- | --- | --- |
| Cape Russell | *I. cordata* | 3 | 17 | Feb 2004 |
| Cape Russell | *P. antarctica* | 3 | 17 | Feb 2004 |
| Cape Russell | *P. cartilagineum* | 3 | 15 | Feb 2004 |
| Adélie Cove | *I. cordata* | 3 | 8 | Feb 1990 |
| Adélie Cove | *P. antarctica* | 3 | 8 | Feb 1990 |
| Adélie Cove | *P. cartilagineum* | 3 | 8 | Feb 1990 |
| Adélie Cove | *I. cordata* | 3 | 0.5 | Jan 1994 |
| Adélie Cove | *P. cartilagineum* | 3 | 0.5 | Jan 1994 |
| Adélie Cove | *P. antarctica* | 10 | 10 | Jan 2012 |
| Adélie Cove | *P. cartilagineum* | 3 | 5 | Jan 2012 |
| Adélie Cove | *P. cartilagineum* | 3 | 10 | Jan 2012 |
| Adélie Cove | *P. cartilagineum* | 3 | 15 | Jan 2012 |
| Adélie Cove | *P. cartilagineum* | 3 | 20 | Jan 2012 |
| Faraglione | *I. cordata* | 3 | 2 | Jan 1990 |
| Faraglione | *P. antarctica* | 3 | 12 | Jan 1990 |
| Faraglione | *P. antarctica* | 3 | 16 | Jan 1994 |
| Faraglione | *I. cordata* | 3 | 3 | Jan 1995 |
| Faraglione | *I. cordata* | 3 | 5 | Jan 1998 |
| Faraglione | *P. antarctica* | 3 | 5 | Jan 1998 |
| Faraglione | *P. antarctica* | 3 | 15 | Jan 1998 |
| Faraglione | *I. cordata* | 3 | 5 | Feb 2000 |
| Faraglione | *P. antarctica* | 3 | 15 | Feb 2000 |
| Faraglione | *I. cordata* | 3 | 6 | Feb 2001 |
| Faraglione | *I. cordata* | 3 | 8 | Feb 2001 |
| Faraglione | *P. antarctica* | 3 | 8 | Feb 2001 |
| Faraglione | *P. antarctica* | 3 | 13 | Feb 2001 |
| Faraglione | *P. antarctica* | 3 | 15 | Jan 2001 |
| Faraglione | *P. antarctica* | 10 | 10 | Jan 2012 |
| Faraglione | *P. cartilagineum* | 3 | 10 | Jan 2012 |
| Molo | *I. cordata* | 3 | 5 | Feb 1994 |
| Molo | *I. cordata* | 3 | 5 | Feb 1998 |
| Molo | *I. cordata* | 3 | 5 | Jan 2000 |
| Molo | *I. cordata* | 3 | 6 | Feb 2001 |
| Molo | *P. antarctica* | 3 | 6 | Feb 2001 |
| Molo | *P. antarctica* | 3 | 8 | Feb 2001 |
| Molo | *P. antarctica* | 3 | 12 | Jan 2001 |
| Molo | *P. antarctica* | 3 | 15 | Feb 2001 |
| Molo | *P. antarctica* | 3 | 12 | Dec 2002 |
| Molo | *P. antarctica* | 3 | 17 | Dec 2002 |
| Tethys Bay | *P. antarctica* | 3 | 6 | Jan 1990 |
| Tethys Bay | *I. cordata* | 3 | 7 | Dec 2002 |
| Tethys Bay | *P. antarctica* | 3 | 7 | Dec 2002 |
| Tethys Bay | *P. antarctica* | 3 | 10.5 | Dec 2002 |
| Tethys Bay | *P. antarctica* | 3 | 13.5 | Dec 2002 |
| Tethys Bay | *P. antarctica* | 10 | 10 | Jan 2012 |
| Cape Evans | *P. antarctica* | 10 | 10 | Jan 2011 |

**S2 Table** Average abundance of diatoms associated with *Phyllophora antarctica* and *Plocamium cartilagineum*, and their contribution to the dissimilarity found between the groups.

|  | Average abundance | | Average dissimilarity | Contribution (%) | Cumulated (%) |
| --- | --- | --- | --- | --- | --- |
|  | *Phyllophora* | *Plocamium* |  |  |  |
| *Navicula perminuta* | 20.19 | 25.57 | 6.28 | 11.71 | 11.71 |
| *Cocconeis fasciolata* | 18.08 | 13.19 | 3.08 | 5.57 | 17.46 |
| *Fragilariopsis nana* | 6.97 | 9.96 | 2.98 | 5.56 | 23.01 |
| *Achnanthes vicentii* | 8.3 | 11.49 | 2.96 | 5.52 | 28.54 |
| *Pseudogomphonema kamtschaticum* | 3.69 | 5.94 | 1.89 | 3.51 | 32.05 |
| *Tabularia tabulata* | 2.76 | 4.73 | 1.78 | 3.32 | 35.37 |

**S3 Table** Average abundance of diatoms associated with *Phyllophora antarctica* and *Iridaea cordata*, and their contribution to the dissimilarity found between the groups.

|  | Average abundance | | Average dissimilarity | Contribution (%) | Cumulated (%) |
| --- | --- | --- | --- | --- | --- |
|  | *Phyllophora* | *Iridaea* |  |  |  |
| *Cocconeis fasciolata* | 18.08 | 3.76 | 9.55 | 14.23 | 14.23 |
| *Navicula perminuta* | 20.19 | 17.96 | 9.17 | 13.68 | 27.91 |
| *Achnanthes vicentii* | 8.3 | 2.7 | 3.74 | 5.57 | 33.48 |
| *Fragilariopsis nana* | 6.97 | 3.83 | 3.59 | 5.36 | 38.84 |
| *Cocconeis antiqua* | 1.79 | 6.76 | 3.43 | 5.12 | 43.96 |
| *Pseudogomphonema kamtschaticum* | 3.69 | 4.28 | 2.57 | 3.82 | 47.78 |

**S4 Table** Average abundance of diatoms associated with *Plocamium cartilagineum* and *Iridaea cordata*, and their contribution to the dissimilarity found between the groups.

|  | Average abundance | | Average dissimilarity | Contribution (%) | Cumulated (%) |
| --- | --- | --- | --- | --- | --- |
|  | *Plocamium* | *Iridaea* |  |  |  |
| *Navicula perminuta* | 25.57 | 17.96 | 8.6 | 13.35 | 13.35 |
| *Cocconeis fasciolata* | 13.19 | 3.76 | 5.73 | 8.88 | 22.23 |
| *Achnanthes vicentii* | 11.49 | 2.7 | 4.72 | 7.32 | 29.55 |
| *Fragilariopsis nana* | 9.96 | 3.83 | 4.11 | 6.38 | 35.93 |
| *Pseudogomphonema kamtschaticum* | 5.94 | 4.28 | 2.79 | 4.33 | 40.26 |
| *Tabularia tabulata* | 4.73 | 0.03 | 2.2 | 3.41 | 43.67 |
